# Health effects of different dietary iron intakes: a systematic literature review for the 5th Nordic Nutrition Recommendations

**DOI:** 10.3402/fnr.v57i0.21667

**Published:** 2013-07-12

**Authors:** Magnus Domellöf, Inga Thorsdottir, Ketil Thorstensen

**Affiliations:** 1 Department of Clinical Sciences, Pediatrics, Umeå University, Umeå, Sweden; 2 Unit for Nutrition Research, School of Health Sciences, University of Iceland and Landspitali National University Hospital of Iceland, Reykjavik, Iceland; 3 Department of Medical Biochemistry, St Olavs Hospital, Trondheim University Hospital, Trondheim, Norway

**Keywords:** iron, iron deficiency, anemia, child development, pregnancy, diabetes

## Abstract

**Background:**

The present literature review is part of the NNR5 project with the aim of reviewing and updating the scientific basis of the 4th edition of the Nordic Nutrition Recommendations (NNR) issued in 2004.

**Objective:**

The objective of this systematic literature review was to assess the health effects of different intakes of iron, at different life stages (infants, children, adolescents, adults, elderly, and during pregnancy and lactation), in order to estimate the requirement for adequate growth, development, and maintenance of health.

**Methods:**

The initial literature search resulted in 1,076 abstracts. Out of those, 276 papers were identified as potentially relevant. Of those, 49 were considered relevant and were quality assessed (A, B, or C). An additional search on iron and diabetes yielded six articles that were quality assessed. Thus, a total of 55 articles were evaluated. The grade of evidence was classified as convincing (grade 1), probable (grade 2), suggestive (grade 3), and inconclusive (grade 4).

**Results:**

There is suggestive evidence that prevention or treatment of iron deficiency (ID) and iron deficiency anemia (IDA) improves cognitive, motoric, and behavioral development in young children, and that treatment of IDA improves attention and concentration in school children and adult women. There is insufficient evidence to show negative health effects of iron intakes in doses suggested by the NNR 4. There is insufficient evidence to suggest that normal birth weight, healthy, exclusively breast-fed infants need additional dietary iron before 6 months of life in the Nordic countries.

An iron concentration of 4–8 mg/L in infant formulas seems to be safe and effective for normal birth weight infants. There is probable evidence that iron supplements (1–2 mg/kg/day) given up to 6 months of age to infants with low birth weight (<2,500 g) prevents IDA and possibly reduce the risk of behavioral problems later on. There is probable evidence that ID and IDA in pregnant women can be effectively prevented by iron supplementation at a dose of 40 mg/day from week 18–20 of gestation. There is probable evidence that a high intake of heme iron, but not total dietary, non-heme or supplemental iron, is associated with increased risk of type 2 diabetes (T2D) and gestational diabetes.

**Conclusions:**

Overall, the evidence does not support a change of the iron intakes recommended in the NNR 4. However, one could consider adding recommendations for infants below 6 months of age, low birth weight infants and pregnant women.

Iron is essential not only for hemoglobin (Hb) production and oxygen transport but also for many enzymes involved in, e.g. cellular energy metabolism. Mild iron deficiency (ID) will lead to depleted iron stores, usually defined as a low serum ferritin (s-Ft). More severe ID will lead to iron deficiency anemia (IDA). Globally, IDA is the most common micronutrient deficiency with about 600 million affected (1). ID and IDA have been associated with poor physical, cognitive and behavioral performances, impaired neurodevelopment in children as well as growth inhibition, hypertension and reduced immune function (2–5).

Iron is unique among nutrients because the human body has no mechanism for iron excretion and excessive iron intakes can therefore result in iron overload if intestinal iron absorption is not sufficiently down-regulated. Since iron absorption is homeostatically regulated, at least in adults, the risk of iron overload from dietary iron is mainly limited to individuals with hereditary hemochromatosis, a relatively common disorder in the Nordic countries, with a reported frequency of homozygosity for the C282Y mutation ranging from 0.20 to 0.75% (6–8). However, high dietary iron intakes may have adverse effects even in the absence of iron overload, since iron is a very reactive element and a potent pro-oxidant. High iron intakes and high serum ferritin concentrations have been suggested to be associated with poor growth, cancer, cardiovascular disease, infections, and diabetes (9–13).

Iron requirements are very different depending on age and gender. Individuals at highest risk of ID are young children and fertile women. Due to the normal iron endowment at birth, healthy, term, normal birth weight infants are theoretically virtually independent on external iron during the first 6 months of life. However, after that age, infants and toddlers have very high dietary iron requirements (about 1 mg/kg/day) due their rapid growth. Breast milk as well as cow's milk has a low iron content so young children need iron-rich complementary foods or iron supplements to achieve these intakes.

The other risk group for ID is fertile women, primarily due to their iron losses associated with menstruation. Furthermore, it is believed that women are adapted to a lower iron status than men. Indeed, in contrast to other biomarkers for nutrition, there are gender-specific cut-offs for the definition of ID and IDA, allowing lower iron status markers in fertile women. During pregnancy, iron requirements are increased due to the iron needs of the growing fetus.

ID is usually defined using one or several of the available markers of iron status, including serum ferritin, transferrin saturation, soluble transferrin receptors, zinc protoporphyrin, mean cell volume, red cell distribution width, reticulocyte Hb, and hepcidin. Serum ferritin is the most commonly used marker of iron status and has been shown to closely parallel the size of body iron stores in adults as measured by bone marrow staining or repeated phlebotomy. However, it is important to note that serum ferritin will be elevated in a state of infection, inflammation or liver disease, limiting the usefulness of serum ferritin for diagnosis of ID in those cases.

In the 4th edition of Nordic Nutrition Recommendations (NNR) issued in 2004 (NNR 2004), the recommended daily intake (RDI) of iron was set to 8 mg/day for children from 6 months to 5 years of age, 9 mg/day for children 6–9 years, 11 mg/day for 10–17-year old boys and for 10–13-year old girls, 15 mg/day for 14–17 year old girls and for women of childbearing age, including lactating women, and 9 mg/day for adult men and post-menopausal women. Even though the NNR 2004 stated that the physiological need of some women for iron cannot be satisfied during the last two thirds of pregnancy with foods only and that supplemental iron is needed, the NNR 2004 did not give a specific recommendation for iron intake for pregnant women. Furthermore, the NNR 2004 did not give recommendations for iron intake for infants below 6 months of age.

The present systematic literature review is a part of the NNR5 project with the aim of reviewing and updating the scientific basis of the 4th edition of the NNR issued in 2004 (Nord 2004:13). A number of systematic literature reviews will form the basis for establishment of dietary reference values in the 5th edition of NNR.

## Aims

The overall aim was to review recent scientific data on health effects of different dietary iron intakes at different life stages (infants, children, adolescents, adults, elderly, and during pregnancy and lactation), in order to estimate the requirements for adequate growth, development, and maintenance of health.

## Research questions

Two specific research questions were developed:

1. What is the minimal dose of dietary iron intake that will prevent poor functional or health outcomes in different age groups within the general population including the risk groups for ID?
2. What is the highest dose of dietary iron intake that is not associated with poor functional or health outcomes in different age groups within the general population including some risk groups for iron overload?

Dietary iron intake was defined as intake for at least 6 months (3 months for infants) from dietary sources or iron supplements. However, some of the included intervention studies were designed with shorter exposure time.

The main functional or clinical outcomes of interest were anemia, cognitive/behavioural function, growth and development, and adverse effects, including the possible risk of cancer and cardiovascular disease.

## Methods

Search terms were defined during 2010, in collaboration with Jannes Engqvist, librarian at the Swedish National Food Agency. The search terms are presented in Appendix 1. In addition, the six papers not included in the original search were found by searching through reference lists of relevant papers known to the authors. Thus, the final search resulted in 1,076 abstracts. Studies published from January 2000 until September 2010 where included. Abstract screening was conducted according to the guidelines for Systematic Literature Reviews for the 5th edition of the NNR. Inclusion criteria in the abstract screening process were the following: Nordic or English language, study population relevant for Nordic countries (excluding populations from low- or middle-income countries in Africa, South America, and Asia), study in healthy humans of relevance to the research questions, full paper (excluding letters, news articles, and congress reports). Of the 1,076 abstracts, 276 fulfilled these criteria and were ordered as full-text articles. Each full text article was screened by two of us and additional papers were excluded according to the following criteria: effect of iron intake not measured, unhealthy population, no health outcome reported, general (non-systematic) review, iron intake from both diet and supplement not assessed. After this exclusion, 49 articles remained and were quality assessed. The full exclusion history is provided in Appendix 2. The selected papers were grouped according to clinical outcomes and different age stages into the following categories:

Anemia and iron status outcomes (specifically iron status in fertile women, pregnant women, and infants), physical performance, cognition and behavior, fetal and child growth, interactions with other food components (including tea, zinc, and copper), adverse effects (including cancer, cardiovascular disease, gastrointestinal effects, hypospadia, Barrett′s esophagus, rheumatoid arthritis, and type 2 diabetes [T2D]).

To ensure the inclusion of the most recent evidence, a complementary search was conducted at the end of January 2012 covering the period between the first search until the end of December 2011. This search resulted in 187 abstracts. Following the same procedure as in the main search, 79 and 61 abstracts were excluded during two rounds of evaluation according to the criteria of Level 1–3 (Appendix 2). After this, 47 abstracts remained and were obtained as full text papers. Quality assessment according to Level 4 (Appendix 2) was performed on papers regarded as particularly important by the expert group. After a thorough systematic review of papers relevant for each outcome, the evidence was graded as either convincing (grade 1), probable (grade 2), suggestive (grade 3) or no conclusion.

The term ‘diabetes’ was unintentionally lacking in the original search. When this was discovered (June 2012), a simple supplementary search in PubMed using the term ‘dietary iron diabetes’ was performed. This produced 132 abstracts for the period 1 January 2000–31 December 2011. Following the same procedure as in the main search, 124 abstracts were excluded. The remaining eight abstracts were obtained as full text articles, and were evaluated with the Quality Assessment Tool (QAT). This resulted in the exclusion of another two articles, leaving six studies on the association between dietary iron intake and diabetes. Thus, a total of 55 articles (49 from the original search, six from the diabetes search) were evaluated using the QAT.

## Results

To evaluate the quality of the selected articles (*N*=55), we used the QAT received from the NNR5 secretary. The QAT included questions about study design, recruitment, compliance, dietary assessment, confounders, statistics, outcomes, etc. The summary of findings from studies graded as A or B according to QAT is presented in Summary Tables 1–11. Main results of the papers graded C are given in the text, but those studies are not used in the final grading of evidence.

## Anemia and iron status

### Infants and young children

Iron supplements are usually not recommended for healthy, breast-fed infants. However, two recent studies have examined the effects of iron supplements (7–7.5 mg/day, corresponding to 1–2 mg/kg/day) given to term, normal birth weight breast-fed infants from 1 to 6 months of age. In the study by Friel et al. (study quality B), a significant effect was observed in Hb at 6 months but no remaining effect at 12 months (14). A weakness of this study was low power and a high attrition rate. In the study by Ziegler et al. (study quality B), a transient effect on s-Ft was observed at 6 months, which no longer remained at 9 months (15). In addition, we included 2 randomized controlled trials (RCTs) of iron supplements given to breast-fed infants from 4 months of age. In the study by Yurdakök et al. (study quality B), no effect was observed on iron status at 7 months after giving iron supplements (1 mg/kg/day or 7 mg/kg/week) (16). In the study by Domellöf et al. (study quality A), a significant effect on iron status, including Hb, was observed in Swedish infants receiving iron supplements (1 mg/kg/day) from 4 to 9 months of age, but there was no reduction in the already-low prevalence of IDA (17). Taken together, these four studies give probable evidence (grade 2) that iron supplementation of breast-fed infants results in a transient increase in iron status (Hb or s-Ft). However, since no reduction in IDA was observed, the data do not support that term, normal birth weight, healthy, breast-fed infants need additional dietary iron before 6 months of life in populations with a low general prevalence of IDA such as the Nordic countries.

In a study by Hernell and Lönnerdal (study quality B), term, normal birth weight infants were randomized to an infant formula with 2 or 4 mg Fe/L from 1 to 6 months of age (18). No significant difference in iron status was observed at 6 months. However, this was a very small study with only 10–12 infants in each group so it was not powered to study effects on ID or IDA. Based on this study, no conclusive evidence (grade 4) can be made regarding whether an iron concentration of 2 mg Fe/L infant formula is sufficient to prevent ID.

We included two cohort studies investigating iron intakes and iron status in Icelandic and New Zealand children (19, 20). Gunnarsson et al. (study quality A) measured iron intake and iron status in 70 Icelandic 2-year-olds and found low s-Ft concentrations (<12 µg/L) in 27%, an ID prevalence of 9% (defined as s-Ft<12 µg/L and mean corpuscular volume (MCV) <74 fL), and only one case of IDA (ID and Hb<105 g/L). Mean (SD) iron intake in that study was 7.57 (4.2) mg/day, which is slightly lower than the NNR4 recommendation of 8 mg/day. Interestingly, Gunnarsson et al. found no significant correlation between iron intakes and iron status in this study but found that a high intake of cow's milk (≥450 mL/day) was significantly associated with ID (19). Heath et al. (study quality B) investigated iron intakes and iron status in a cohort of 74 New Zealand children followed from 9 to 24 months of age (55 remained at 24 months) (20). At 9 months of age, median iron intake was 7.0 mg/day, which is slightly lower than the NNR4 recommendation of 8 mg/day. However, at 12–24 months, median iron intakes were 4.3–4.9 mg/day. Still, the prevalence of IDA in this cohort was relatively low: 7% at 9–18 months of age and no case of IDA was found at 24 months. In this study, IDA was defined as Hb<110 g/L and MCV<77 fL, i.e. higher cut-offs than the Icelandic study and, unusually, s-Ft was not included in the definition of IDA. The correlation between iron intakes and iron status was not reported in this study.

In conclusion, both of these studies report a lower iron intake than recommended in 9–24 month-old children but the prevalence of IDA was low at 24 months of age. The finding of a significant association between cow's milk intake >500 ml/day and ID in the Icelandic study supports previous Nordic data suggesting that a high cow's milk intake (in that study >300 mL/day) is associated with an increased risk of ID in toddlers.

### Fertile women

The original search returned no articles examining the dietary intake of iron and iron status in free-living, iron-replete women of childbearing age.

One small study (study quality B) examined iron status markers in 25 iron deficient women consuming an oily fish or a red meat diet, in a randomized crossover dietary intervention study of two 8-week periods (21). At inclusion, the women (aged 18–30 years) were menstruating, non-smoking, non-anemic (Hb>11 g/dL), had low iron stores (Ft<30 µg/L), had not used iron supplements for the last 12 months, had no chronic gastric or iron-metabolism related disease, had no fish allergy, or were not vegetarian. The average dietary iron intake was 11.54 mg/day during the oily fish diet, and 13.93 mg/day during the red meat diet, both diets providing a lower daily iron intake than the NNR4 recommendation for menstruating women (15 mg/day). The study found a non-significant increase in s-Ft and a non-significant decrease in serum transferrin receptors (sTfR) during the red meat diet, and opposite, non-significant changes during the oily fish diet. The authors concluded that an oily fish diet does not decrease iron status in iron deficient women during 8 weeks compared to a red meat diet.

The complementary search yielded two studies that addressed iron status of iron deficient fertile women. One was a Cochrane review, whereas the other was an intervention study examining the effect of microencapsulated iron-pyrophosphate-fortified fruit juice (22, 23).

The Cochrane review examined the effect of intermittent iron supplementation versus no intervention or placebo. However, of the 21 studies included, only one, small study (*N*=24) was on a population relevant to NNR5 (24). The authors conclude, in line with the general conclusion of the Cochrane review, that daily supplementation with 50 mg Fe can provide a better correction of ID than a twice weekly supplementation of 50 mg Fe for 3 months in young women with low s-Ft levels.

The intervention study lasted for 16 weeks and supplied 18 mg Fe/day as microencapsulated iron-pyrophosphate in 500 mL fruit juice (Fe group *N*=58), or fruit juice only (placebo group, *N*=64) to women aged 18–35 years, non-smoker, non-pregnant, non-breastfeeding, with low iron stores, defined as s-Ft<40 ng/mL and Hb >110 g/L (23). At baseline, the average daily iron intake was 15.1 mg in the placebo group and 13.7 mg in the Fe group. During the 16 weeks, the Fe group had an average iron intake of 30.4 mg/day, whereas the intake of the placebo group actually declined to 12.9 mg Fe/day. The mean s-Ft value of the placebo group remained constant over the intervention period (Start: 26.9 ng/mL, End: 22.8 ng/mL), whereas the mean s-Ft in the Fe group increased significantly from 25.4 to 40.7 ng/mL. The authors conclude that their results suggest that iron-fortified fruit juice could be used as dietary treatment to effectively correct anemia. The study shows that daily intake of an iron-enriched fruit juice results in increased s-Ft concentrations in healthy, non-anemic menstruating women. However, the study does not show that the additional iron reduces the prevalence of ID or IDA. Thus, the available literature present no new information and allow no new conclusions to be drawn regarding any health outcomes following high or low dietary iron intake by healthy fertile women.

### Pregnant women

We found a total of seven studies that examined various aspects of the association of iron supplement intake and anemia in pregnancy. Two studies examined the effect of supplementing with a low iron dose from week 18–20 of gestation to delivery or 8 weeks postpartum (25, 26). Two studies investigated the benefit of adjusting iron supplementation based on s-Ft values in early pregnancy (27, 28). One study examined short term effect of iron supplementation from week 20 to 28 of gestation (29), one study looked at healthy, iron sufficient adolescent and adult women receiving iron from week 10–16 of gestation until term (30), and another study looked at the effect of iron supplementation from day 4 after delivery to the end of the 3rd month postpartum (31).

Milman et al. (study quality B) followed 427 women from 18 weeks of gestation to 8 weeks postpartum (25). The participants were randomly assigned to one of four groups receiving 20, 40, 60, or 80 mg Fe/day as ferrous sulphate. Compliance was >90%. The frequency of ID (defined as Ft<13 µg/L) or IDA (defined as ID with Hb<106 g/L at 18 weeks, Hb<105 g/L at 32 weeks, Hb<115 g/L at 39 weeks, Hb<123 g/L at 8 weeks postpartum) at inclusion did not differ between the groups. However, at 32 weeks of gestation the frequency of ID in the 20 mg Fe group (50%) was significantly higher than the frequency in the 40 mg (26%), the 60 mg (17%) and the 80 mg group (13%). Similar, significant differences were found at 39 weeks of gestation (29, 11, 10, and 9%). At 8 weeks postpartum, no statistically significant differences in ID frequencies were found (6.7, 2.9, 2.1 and 0%). The prevalence of IDA at 32 weeks was not different between the four groups. At 39 weeks, the prevalence in the 20 mg Fe group (10%) was higher than the other three groups (4.5, 0, and 1.5%). At 8 weeks postpartum, the prevalence of IDA was low and equal in the four groups (0, 0, 2.1 and 0%, respectively).

The authors conclude that in Danish women supplementation with 40 mg Fe/day from 18 weeks of gestation prevents ID in 90% and IDA in at least 95% of the women during pregnancy.

Makrides et al. (study quality A) performed a similar study on 386 Australian women (26). From week 20 of gestation until delivery, participants were randomly assigned to receive either 20 mg Fe/day (as ferrous sulphate), or placebo. Compliance was 85–86%. Iron status was examined at delivery and at 6 months postpartum. At delivery, the frequency of IDA (defined as Ft<12 µg/L and Hb<110 g/L from week 20 until delivery, or defined as Ft<15 µg/L and Hb<120 g/L at 6 months postpartum) in the iron group was 3% compared to 11% in the placebo group Relative Risk (RR) was 0.28 (95% CI: 0.12–0.68; *P*<0.005). At 6 months postpartum there was no difference in IDA frequency between the two groups. The frequency of ID at delivery was 35% in the iron group and 58% in the placebo group (RR=0.60; 95% CI: 0.48–0.76; *P*<0.001), and at 6 months postpartum the frequencies were 16% and 29% (RR=0.57; 95% CI: 0.38–0.84; *P*<0.005) in the two groups.

The authors conclude that supplementation with 20 mg Fe/day from week 20 of gestation is an effective strategy for preventing IDA and ID.

Milman et al. (25) did not actually measure iron intake from foods, but give a reference to the average iron intake of Danish women of 8.5–9.1 mg Fe/day (32). Makrides et al. using a food frequency questionnaire (FFQ), found an average intake of iron from foods of approximately 14 mg/day (26). Thus, an iron supplement dose of 20 mg/day as used by Makrides et al. would exceed the US recommendation (33) for dietary iron of 27 mg/day 1.25–fold, whereas the 40 mg Fe/day dose suggested by Milman et al. is 1.8-fold higher.

Taken together, these two studies strongly suggest that supplementation with 20–40 mg Fe/day (corresponding to a total iron intake of 34–49 mg/day) from week 18–20 of gestation would be effective in preventing ID and IDA during pregnancy This conclusion is also supported by the two studies of Sandstad et al. (study quality B), and Milman et al. (study quality B) in which iron supplementation was adjusted according to s-Ft concentrations in early pregnancy (27, 32). Sandstad et al. compared the effect of giving advice only (Group I) with giving advice in combination with iron supplementation (Group II), on iron status 6 weeks postpartum (27). They found no differences between the two groups, i.e. giving advice only works just as well as actively giving the women iron supplements. Through the design of the study, Group II was split into Group IIa (receiving 60 mg Fe/day as ferrous sulphate) and IIb (receiving 27.6 mg Fe/day as 24 mg Fe in the form of ferrous fumarate and 3.6 mg Fe in the form of heme iron). Group IIa had significantly higher s-Ft at 6 weeks postpartum compared to Group IIb (*P*<0.05), and the increase in s-Ft from early pregnancy until 6 weeks postpartum was significantly higher in IIa compared to IIb (*P*<0.04). The prevalence of empty iron stores, defined as s-Ft<12 µg/L, at 6 weeks postpartum was 4.8%. The authors conclude that iron supplementation based on iron status in early pregnancy resulted in adequate iron stores at 6 weeks postpartum. However, the main indicator for iron status at 6 weeks postpartum was peripartum blood loss.

Milman et al. allocated 301 healthy, pregnant Danish women into four groups receiving 20, 40, 60 or 80 mg Fe/day, respectively, from week 18 of gestation until 8 weeks postpartum (28). The participants in the four iron groups were stratified according to their s-Ft concentrations. At inclusion 50.7% had s-Ft ≤ 30 µg/L, 37.7% had s-Ft 30–70 µg/L, and 11.6% had s-Ft>70 µg/L. At delivery women with s-Ft ≤ 30 µg/L in early pregnancy had ID frequencies of approximately 27, 17, 7 and 7% in the 20, 40, 60 and 80 mg Fe/day groups, respectively (*P*<0.001). Women with s-Ft between 30 and 70 µg/L in early pregnancy had ID frequencies of approximately 23, 0, 11 and 4% in the four iron groups (*P*<0.001). Women with s-Ft>70 µg/L at inclusion had no ID. The frequency of IDA at delivery was low, and none of the women with s-Ft>30 µg/L had IDA. The authors suggest that to prevent ID, supplementation with 80–100 mg Fe/day to women with s-Ft ≤ 30 µg/L in early pregnancy, and 40 mg Fe/day for women with s-Ft between 30 and 70 µg/L, is sufficient. There is no need for iron supplement in women with s-Ft>70 µg/L.

Taken together, these two studies suggest that adjusting iron supplementation on an individual basis according to s-Ft values in early pregnancy may be of some benefit. Those with s-Ft below 20–30 µg/L would benefit from receiving an iron dose of 40–60 mg Fe/day, and iron-replete women (s-Ft>60–70 µg/L) would not have to take iron supplements at all.

The data in the four studies above present probable evidence (grade 2) showing that if we want to prevent ID in more than 90% and IDA in more than 95% of women at delivery and at 6–8 weeks postpartum, supplementation with 40 mg Fe/day from week 18–20 of gestation will achieve this. The same supplementation regime would prevent ID in 83% of women having s-Ft<30 µg/L in early pregnancy, and in 100% of women having s-Ft between 30 and 70 µg/L in early pregnancy. At 6–8 weeks postpartum ID and IDA would be prevented in 100% of women. For women with Ft>70 µg/L in early pregnancy, iron supplementation would normally be unnecessary.

As part of a study on birth weight, Cogswell et al. (study quality B) also measured iron status in 275 pregnant, iron sufficient women (Hb>110 g/L and s-Ft>20 µg/L), randomly assigned to receive iron supplements (30 mg Fe/day) or placebo from week 20 to week 28 of gestation (29). They found no differences between iron and placebo with regard to the proportion of women with anemia, low iron stores, or IDA. After adjustment for pre-pregnant weight and log initial s-Ft the prevalence of absent iron stores and IDA was significantly lower in the iron-supplemented group compared to the placebo group.

Meier et al. (study quality C) examined 111 healthy, iron sufficient (median s-Ft>30 µg/L), pregnant adolescent (15–18 years) or adult (>19 years) women, randomly assigned to receive placebo or iron supplementation (60 mg Fe/day) from week 10–16 of gestation until delivery (30). Compliance as measured by pill count, was 84–98%. Overall, 47% of the placebo group and 16% of the iron group exhibited IDA (*P*<0.001). Among the adolescents 59% of the placebo group and 20% of the iron group exhibited IDA (*P*=0.021) at delivery. Median Hb and s-Ft values in adolescents were significantly higher at term in the iron group 1. In adults s-Ft (but not Hb) was significantly higher at second trimester as well as at term in the iron group compared to the placebo group.

The study by Mara et al. (study quality C) examined the effect of supplementing with 80 mg Fe/day (as the commercial product Tardyferon) either alone (*N*=20) or in combination with 0.35 mg folic acid/day (Tardyferon Fol) (*N*=20) starting at 4 days after delivery and continuing until the end of the 3rd month postpartum (31). A control group (*N*=20) received no supplement. At delivery a total of 61.7% of the women were anemic, defined as Hb<11 g/dL. At 1 month postpartum, 15% of women receiving iron alone were anemic compared to 50% in the control group (*P*=0.041, Fisher's exact test, authors’ calculation). At 2 months postpartum, 10% of the Fe group was anemic compared to 35% of the control group (*P*=0.127, Fisher's exact test, authors’ calculation). At 3 months postpartum, only one woman in the control group remained anemic. The authors recommend flat administration of 80 mg Fe/day to all women for at least 3 months after delivery. In our opinion, this small study shows that 80 mg Fe/day, at best, reduces the frequency of anemia during the 1st month postpartum. After 2–3 months, there is no difference between the group receiving iron and the control group. Thus, the study data does not support the recommendations given by the authors.

In addition, a systematic review and meta-analysis was published in 2009 covering the effects of preventive oral iron supplementation during pregnancy (34). The review considered 49 publications published between 1958 and 2008, and two of the studies included in the current review (26, 33). were among the 49 articles covered by the review. Unfortunately, most of the studies in the meta-analysis are published before year 2000, or are performed in populations not relevant to NNR. Furthermore, information on iron dosage and/or food iron intake is missing. In general, however, the seven studies included in our analysis are in line with the results reported in the meta-analysis.

### Effects on offspring of iron supplementation of pregnant women

Iron supplementation of pregnant women has been suggested to improve iron status in the newborn. However, iron transport to the fetus is an active process and the fetus may be protected from ID even when the mother has mild or moderate IDA. This review includes one RCT by Zhou et al. (study quality A), evaluating the effect of 20 mg Fe/day versus placebo given to pregnant Australian mothers from mid-gestation until delivery (35). No significant effect was observed with regard to offspring iron status at 6 months or 4 years of age. This is supported by the systematic review by Pena-Rosas (34) (study quality A) which concludes that there is a lack of convincing evidence that iron supplementation during pregnancy improves infant iron status, even though this was suggested by a single study performed in an Nigerian population with a very high prevalence of IDA (36). In summary, there is no conclusive evidence (grade 4) that iron supplementation of pregnant women in a Scandinavian setting would improve infant iron status. See Summary Table 1.

## Physical performance

Two studies have examined the effect of iron on physical performance in healthy individuals. Unfortunately, none of the studies give any quantitative data (e.g. RR or odds ratio [OR]) on the impact of iron on physical performance.

McClung et al. (study quality B) measured the effect of iron supplementation on 2-mile run time (main physical performance outcome) in female soldiers during basic combat training (BCT) (37). The study was an 8-week randomized, double-blind, placebo-controlled trial (evidence quality B), where the intervention group (*N*=86) received 15 mg Fe/day as Fe(II)-sulphate. Iron intake from foods was estimated, but not measured, to a minimum of 3 mg/day. This was the only iron intake in the placebo group (*N*=85). Approximately 16 and 21% of the participants presented with ID or IDA, respectively, at baseline. The number of individuals with ID or IDA was equally distributed between the intervention group and the placebo group. The main biochemical finding was that iron supplementation attenuated the decline in iron status observed during BCT. This effect was relatively larger in individuals with ID or IDA at baseline. The 2-mile run time post-BCT did not differ between the iron group and the placebo group in iron-replete or ID participants. However, in individuals with IDA at baseline the placebo group had a significantly longer run time compared to all other groups. Furthermore, the placebo group run time of the IDA participants was on average 110 sec longer than the IDA iron group. Thus, physical performance of IDA individuals after receiving iron supplements during 8 weeks BCT is similar to that of iron-replete individuals. Iron supplementation to iron-replete or ID individuals has apparently no effect on physical performance, at least not when assessed by 2-mile run time.

The second study (study quality C) was performed on adolescent swimmers (21 male, 21 female) aged 12–17 years and without ID or IDA, during a 6-month training season (38). The participants were equally allocated to three groups of similar age, performance level, and iron status. Group A followed their ordinary diet, but received 47 mg Fe/day as supplement (average total iron intake of 58 mg/day). Group B followed a diet plan rich in iron (average total iron intake of 26 mg/day), whereas Group C followed their normal diet (average total iron intake of 12 mg/day). The groups were followed during the different phases of the training season, and physical performance was assessed by various swimming tests. Although significant fluctuations in iron status during the training season could be observed, no significant differences in iron status or performance between the three groups could be found. The authors conclude that ‘iron status and performance of healthy adolescent swimmers were affected by training irrespective of iron intake ranging from one to over five times the RDA over a period of 6 months’.

Taken together, these two studies yield inconclusive evidence (grade 4) that iron supplementation increases physical performance in iron-replete individuals, but limited – suggestive evidence that iron supplementation enhances physical performance in individuals with IDA.

## Cognitive and behavioral function

### Effects on the offspring of iron given to the pregnant mother

This review includes two publications (both study quality A) from the same Australian RCT assessing the effects of iron supplementation during pregnancy on offspring cognitive/behavioral function (39, 40). These studies show that iron supplements (20 mg/day) during the last half of gestation did not have any effect on cognitive function at 4 years or on behavioral scores at 6–8 years in the offspring. The studies provide no conclusive evidence (grade 4) that iron supplementation during pregnancy improves cognitive or behavioral outcomes in the offspring.

### Effects of iron given to infants and children

Only one included intervention trial was performed in a low-risk population comparable to the Nordic population (14). That trial, performed by Friel et al. (study quality B), investigated the effects of iron supplements (7.5 mg daily; corresponding to 1–2 mg/kg/day) given from 1–6 months of age to healthy, term, normal birth weight, breast-fed Canadian infants and showed significantly better psychomotor development in iron supplemented infants at 12–18 months of age (PDI 100 vs. 93) (14) However, this was a small study with high attrition and only 26 iron supplemented and 20 control infants analysed, leaving the possibility of a type I error.

A recent meta-analysis (study quality A), based on only three RCTs showed that iron supplements to non-anemic infants has a significant positive effect on psychomotor scores but no effect on mental development index at 12 months of age (41). One of these RCTs was the Friel trial described above and the other two were performed in high-risk populations (Indonesia (42) and very low-income Canadian families (43)), making it difficult to translate these results directly to the Nordic population.

## Fetal and child growth

### Fetal growth and pregnancy duration

Iron given to pregnant women has previously been suggested to improve fetal growth and result in higher birth weights. We included one cohort study (study quality B) and one meta-analysis (study quality A) of the effects on fetal growth (34, 44). The cohort study showed that iron intake (from diet and supplements) in pregnant adolescents was not significantly associated with growth restriction (small for gestational age at birth) or preterm birth (44). Similarly, the meta-analysis by Pena-Rosas, including 10 trials and >6,000 subjects, showed that iron supplements during pregnancy had no significant effect on birth weight or the proportion of infants with low birth weight (<2,500 g) (34). Based primarily on the meta-analysis, we judge that there is no conclusive evidence (grade 4) that iron supplements during pregnancy improve fetal growth, or reduce the proportion of children born prematurely, small for gestational age or with low birth weight.

### Child growth

Additional iron given to iron-replete infants has been suggested to impair growth. We included one RCT (study quality A) and one meta-analysis (study quality A) regarding the effects of iron on child growth. The RCT showed that iron supplements at a dose of 1 mg/kg/day from 4–9 or 6–9 months of age significantly impaired longitudinal growth (9). This effect was mainly seen in those Swedish infants who were iron-replete and had a high initial Hb concentration, suggesting that iron supplements may impair growth in iron-replete infants who do not belong to a risk group for IDA. On the other hand, a meta-analysis by Ramakrishnan including 27 trials (most in low-income countries) showed no significant effect of iron supplements on growth in children <5 years of age (45). However, children were not stratified by initial iron status in the meta-analysis. In summary, there is no conclusive evidence (grade 4) that iron supplements impair growth in iron-replete children.

## Hypertension and cardiovascular disease

There were four papers on iron intake and hypertension, and one on iron intake and mortality from cardiovascular disease; all papers were evaluated to be of quality B. See Summary Table 7. Additionally, the recent systematic review of the UK Department of Health was considered (46).

### Hypertension

Two of the studies concerned maternal intake of iron during pregnancy and blood pressure of the offspring aged 3 and 7 years (47, 48). A study by Belfort et al. (47) showed that by each 10 mg increase in iron intake in the first trimester, systolic blood pressure of 3-year-olds increased by 0.4 mm Hg (95% CI: 0.1–0.7) (47). Brion et al. (48) found no effects of maternal total iron intake on child's blood pressure when all confounders were included. Those women who had received iron supplement at any trimester in pregnancy had a significantly lower systolic blood pressure, −0.63 mm Hg (95% CI: −1.09–(−0.17)) (48). Belfort et al. (47) found no effects from mothers‘ iron status in pregnancy on the children‘s blood pressure, but Brion et al. (48) saw a positive association between anemia in early pregnancy and child‘s blood pressure (see Summary Table 7). Two other studies on blood pressure and iron intake in adults were explored, one prospective observational study and one cross sectional study (49, 50). Both of these studies found an inverse association between iron intake and blood pressure (see Summary Table 7). Low non-heme iron intake associated negatively with systolic blood pressure and with hypertension in all models tested in the 5.4 years prospective study. The third tertile of intake of non-heme iron had OR=0.63 (95% CI: 0.41–0.96) compared to the first tertile, i.e. average intake of 13.7 mg/day non-heme iron had 37% lower risk for hypertension than those with 7.7 mg/day on average (49). This study was supported by a number of public and private enterprises, but the authors all declared no conflict of interest. Tzoulaki et al. (50) conducted a multinational cross sectional study and also found a negative association between both dietary total and non-heme iron intake and systolic blood pressure (50). Both studies found no significant association between heme iron and blood pressure, but a positive association between the intake of red meat and blood pressure level (49, 50), but which became insignificant after adjustment for dietary pattern score (49). The evidence is inconclusive (grade 4) regarding any association between iron intake during pregnancy and blood pressure or hypertension in the offspring.

### Cardiovascular disease

Lee et al. analyzed results from the Iowa women's health study cohort (study quality B) for associations between cardiovascular disease mortality and dietary iron, zinc, and alcohol (51). In the total sample, neither heme nor non-heme iron was associated with the risk or cardiovascular disease mortality. The authors hypothesized that alcohol is a trigger of disturbing iron homeostasis creating a redox active non-transferrin form of iron. Heme iron intake associated positively with cardiovascular disease mortality when alcohol intake was above 10 g/day and stronger when it was higher, e.g. above 30 g/day (see Summary Table 7). Highest intake quintile vs. lowest of heme iron gave RR= 2.47(CI: 1.1–5.55) when alcohol intake was above 10 g/day. When alcohol consumption was >30 g/day, significantly higher RR was found for third, fourth, and fifth quintile of heme iron intake compared to the lowest quintile of intake, i.e. RR ranged from 2.62(CI: 1.03–6.67) to 4.11(CI: 1.14–14.87).

The UK Department of Health covered iron and cardiovascular disease in their report on iron and health from 2010 (46). The report discusses the hypothesis of blood loss or decreased body iron stores as protective against cardiovascular disease. The report suggests how iron may play a role in the development of atherosclerosis by promoting oxidation of LDL cholesterol through catalyzing the formation of free radicals or promoting free radical mediated myocardial damage following an ischemic event. The UK report includes five prospective studies on dietary iron intake and cardiovascular disease, which showed a median RR 1.48 of highest vs. lowest heme iron intake, but the report also points out other explanations for this observed association. They found no effect of total iron intake or of body iron as s-Ft levels on cardiovascular disease. Taken together there is no conclusive evidence (grade 4) about an association between iron intake and cardiovascular disease risk even though there is limited but suggestive evidence (grade 3) for increased risk of cardiovascular disease following high heme iron intake among women using more than 30 g of alcohol per day.

## Diabetes mellitus

Our search retrieved seven studies (one in the original search, six in the diabetes search) examining the association between iron intake and the risk of diabetes. Five of these were of study quality B or A, and are listed in Summary Table 8, whereas two of the studies were of quality C and are briefly summarised here. In addition, the complementary search yielded one article.

### Type 2 diabetes

Lee et al. presented a large prospective study on the Iowa women's health study cohort (study quality B) (13). They found RR=1.28 (95% CI: 1.04–1.58) for developing T2D when comparing the 5th quintile of heme iron intake with the 1st quintile during 11 years follow-up. This positive association between heme iron intake and T2D was strongest among those women who consumed >15 g/day of alcohol, even though alcohol intake itself reduced the risk of T2D.

Rajpathak et al. (study quality A) conducted a study within the Nurses’ Health Study cohort and followed 85,000 women for 20 years. During this time period they documented 4,599 incident cases of T2D. Heme iron intake was positively associated with risk; RR=1.28 (95% CI: 1.14–1.45) for developing T2D when comparing the 5th quintile of heme iron intake with the 1st quintile (52). No association between total, dietary, supplemental, or non-heme iron and risk of T2D was found. The authors also state that no effect of alcohol consumption could be found.

Jiang et al. (study quality B) followed 38,394 men for 12 years, participating in the Health Professionals’ Follow-up Study (53). They found that intakes of total heme iron (RR=1.28; 95% CI: 1.02–1.61) and heme iron from red meat (RR=1.63; 95% CI: 1.26–2.10) were associated with an increased risk of T2D. Total iron intake or heme-iron intake, from sources other than red meat, were not associated with risk of T2D.

Thus, the three studies above all indicate heme iron as a risk factor for developing T2D.

This finding is indirectly supported by another, case-control study, by Jiang et al. where they measured s-Ft and s-Ft/Hb ratio, as markers for iron stores, in 698 cases with T2D and 716 controls. They found RR=3.20 (95% CI: 2.22–4.62) for developing T2D when comparing the 5th quintile of s-Ft with the 1st quintile. Inversely, when comparing the 1st quintile of s-Ft/Hb ratio with the 5th quintile, they found RR=2.59 (95% CI: 1.82–3.68). Cases had significantly higher intake of heme iron than controls (54).

Finally, Forouhi et al. (study quality C) investigating 360 clinically incident T2D cases with 758 controls, nested within the EPIC (European Prospective Investigation of Cancer)-Norfolk Study, found that baseline s-Ft values were higher among cases than controls (55). The risk of T2D was significantly elevated in those with clinically increased s-Ft (>300 ng/mL for men, >150 ng/mL for women) compared with the lowest quartile (OR=7.4; 95% CI: 3.5–15.4). Among the participants with clinically raised s-Ft total iron intake was not increased compared with the lowest s-Ft quartile, but red meat intake and alcohol consumption was significantly increased.

### Gestational diabetes

Qiu et al. examined 3,158 pregnant women in a prospective cohort study (study quality B). Approximately 5% (*n*=158) of the women developed gestational diabetes mellitus (GDM) (56) In line with the findings above they found that women reporting the highest heme iron intake (≥ 1.52 mg/day) had an increased risk (RR=3.31; 95% CI: 1.02–10.72) for developing GDM compared to those with the lowest heme iron intake (<0.48 mg/day). Non-heme iron intake was inversely, but not statistically significant, associated with GDM risk. From the complementary search we retrieved a large study on the association of pre-pregnancy dietary iron intake and GDM. Bowers et al. conducted a prospective study among 13,475 pregnant women between 1991 and 2001 in the Nurses’ Health Study II (57). Dietary information was obtained every 4 years through a semiquantitative FFQ. A total of 867 incident cases of GDM were reported during the 10 years. Dietary heme iron intake was significantly associated with GDM. Comparing the 5th quintile of heme iron intake with the 1st quintile, the relative risk of developing GDM was 1.58 (95% CI: 1.21–2.08). No significant associations were observed between total dietary, non-heme, or supplemental iron intake and GDM.

### Type 1 diabetes in children

In a case-control study (study quality C) Ashraf et al. investigated the possible association between iron intake in infants aged 1–4 months and development of type 1 diabetes (T1D) before the age of 6 years (58). They found that median total iron intake (formula, supplement and breast milk) during the first 4 months of life was 1,159 mg in 128 cases and 466 mg in 67 controls (*P*<0.0001). Diabetes cases had significantly shorter breastfeeding duration (4 vs. 12 weeks, *P*=0.001), so the higher iron intake was probably due to intake of iron-fortified infant formula, even though formula intake was not reported in the paper. For each standard deviation increase in iron intake, the OR for T1D was 2.01 (95% CI: 1.183–3.41) among all participants.

## Cancer

Several studies report on the possible association between iron intake and various cancer forms.

### Colon cancer

In two studies (both study quality B), in women, the risk of proximal or distal colon cancer (59), or colorectal adenoma (60), was assessed. The study of Lee et al. was part of the Iowa women's health study and followed a cohort of 34,708 postmenopausal women for 15 years (59). They found no association between dietary heme iron (median intake 1.2 mg/day) or non-heme iron (median intake 11.2 mg/day) and colon cancer. However, the RR for incident distal colon cancer, but not for proximal colon cancer, increased with increasing intake of iron supplements from 0 to 50 mg/day or more, although not statistically significant (*P*_trend_=0.08). In the group with highest iron supplement intake (>50 mg/day), the RR was 1.79 (95% CI: 0.94–3.43). On the other hand, when stratified by intake of fermentable substrates (e.g. insoluble or soluble fibers, or resistant starch), those having an intake of fermentable substrates above median (25.9 g/day), also had a significantly increased RR of 3.78 (95% CI 1.78–7.68, *P*_trend_<0.01). The authors commented however, that since only nine cases were included in the group having a daily intake of iron supplements of >50 mg, the finding of an increased RR could have arisen by chance.

Chan and co-workers went the other way around and compared 527 cases of colorectal adenoma found in the Nurses’ Health Study, with 527 matched controls without colorectal adenoma (60). They examined a number of biochemical iron status parameters and looked for possible associations between iron status and colorectal adenoma. No statistically significant relationship between iron status parameters and the risk of colorectal adenoma could be found.

Taken together these studies do not give any solid evidence supporting the hypothesis that iron is a risk factor for developing cancer of the colon.

The complementary search yielded one additional study on the association between iron and colorectal cancer. Bastide et al. performed a meta-analysis on the relationship between heme iron from meat and the risk of colorectal cancer (61). The analysis contained 5 articles representing 566,607 individuals and 4,734 cases of colon cancer. One of the articles was identical to one of the articles above (59). The relative risk of colon cancer was 1.18 (95% CI: 1.06–1.32) for individuals in the highest category of heme iron intake compared with those in the lowest category.

On the whole, the three studies (50–52) deliver inconclusive evidence (grade 4) regarding the association between iron intake and colorectal cancer (59, 61).

### Lung cancer

Two studies (both study quality B) have examined the possible association between iron and lung cancer. The study of Lee and Jacobs was part of the Iowa Women's Health Study (62). In a cohort of 34,708 postmenopausal women followed for 16 years, 700 lung cancer cases were identified. The authors report no association between total iron intake (dietary iron plus supplements) and lung cancer. On the other hand, dietary heme iron intake was positively associated with lung cancer when subjects were stratified by vitamin C supplements intake. Those with the highest heme iron intake (≥2.44 mg/day) and the highest vitamin C supplement intake (>700 mg/day) had RR=8.97 (95% CI: 1.29–62.51) for lung cancer compared to those with lowest heme iron intake (≤0.77 mg/day). Among current or ex-smokers, the association was even stronger (RR=20.38; 95% CI: 22.50–185.8) and reached statistical significance even in the group having vitamin C supplement intake of >100 mg/day (RR=3.52; 95% CI: 1.24–8.97). The authors suggest that high dietary heme iron intake in combination with high intake of vitamin C supplements may increase the risk of lung cancer in postmenopausal women.

In a case-control (study quality B) study, Zhou and co-workers identified 923 cases of lung cancer at Massachusetts General Hospital over an 8-year period (63). Controls were recruited among healthy friends and non-blood-related family members of patients. Adjusted OR for total iron intake (diet plus supplements) was 2.06 (95% CI: 1.31–3.24; *P*_trend_=0.003), whereas OR for total dietary iron intake was 1.45 (95% CI: 1.03–2.06) when comparing the highest quintile of iron intake with the lowest. In a multivariate model also including dietary zinc and calcium OR increased to 1.95 (95% CI: 1.33–2.86). Furthermore, when compared with subjects in the lowest tertile of iron intake (<11.70 mg/day) and the highest tertile of zinc intake (>11.69 mg/day), subjects who had the highest intake of iron (>14.32 mg/day) and lowest intake of zinc (<9.70 mg/day) had a higher risk of lung cancer (OR=2.01; 95% CI: 0.96–4.2; *P*_trend_ <0.0001). However, in contrast to Lee and Jacobs, this study finds that the increased risk of lung cancer conferred by dietary iron was the result of non-heme iron intake. Heme iron was associated with a decrease in the risk of lung cancer. The adjusted OR for non-heme iron was 1.49 (95% CI: 1.04–2.14; *P*_trend_=0.007) whereas the OR for heme iron was 0.39 (95% CI: 0.28–0.56; *P*_trend_<0.0001).

The complementary search yielded one additional article on iron intake and lung cancer (64). Tasevska et al. examined (among other factors) the effect of heme iron on lung cancer risk among 99,579 participants of the Prostate, Lung, Colorectal and Ovarian Cancer Screening Trial. After up to 8 years of follow-up 782 incident lung cancers (454 male, 328 female) were identified. Heme iron intake had no effect on the risk of lung cancer.

The studies by Lee et al. and Zhou et al. both indicate that iron may be a risk factor for lung cancer (62, 63). However, they reach opposite conclusions on whether heme or non-heme iron is the form of iron conferring the risk. Tasevska et al. found no effect of heme iron (64).

All in all, these three studies deliver inconclusive evidence (grade 4) with regard to the effect of iron intake on lung cancer.

### Breast cancer

A large study on the association between iron intake and breast cancer was performed by Ferrucci and co-workers (study quality B) as part of the Prostate, Lung, Colorectal and Ovarian Cancer Screening Trial (65). The analytical cohort consisted of 52,158 US postmenopausal women. Incident, histologically confirmed, invasive breast cancer cases (*N*=1,205) were identified based on pathology reports and medical records. There was no statistically significant positive association for total iron (diet plus supplements), heme iron, iron from meat, or iron from supplements. However, total dietary iron was positively associated with breast cancer in a dose-response manner. Comparing the fifth to the first quintile of dietary iron intake, the energy adjusted HR for breast cancer was 1.25 (95% CI: 1.02–1.52; *P*_trend_=0.03).

From the complementary search one study addressing the association between iron intake and the risk of breast cancer was found (66). The study used data from the National Institute of Health – AARP Diet and Health study to assess intakes of dietary iron, iron from meat, iron from red meat, and heme iron in relation to breast cancer risk in 116,674 postmenopausal women. During 6.5 years of follow-up, 3,396 cases of invasive breast cancer were identified. Hazard ratios for the highest compared with the lowest quintiles of iron intakes were all close to unity, and there were no increasing trends with increasing iron intakes.

Taken together these two large studies provide no conclusive evidence (grade 4) for an association between iron intake and postmenopausal breast cancer.

### Esophageal cancer

One paper on iron intake and Barrett's Esophagus was evaluated, quality grade B. The study by Corley et al. was a case-control study within the Kaiser Permanente Northern California population (67). The background for the study lies in the hypothesis that high iron stores are a risk factor for esophageal adenocarcinoma. Little human data exists but Corley et al. (67) refer to studies on animals that indicate the association. This study evaluated whether iron intake as well as iron stores were associated with Barrett‘s Esophagus, a metaplastic change that is a strong risk factor for adenocarcinoma. Between 900 and 1,000 individuals were compared as three groups, cases of Barrett's syndrome, gastroesophageal reflux disease patients (GERD) and controls. Intake of dietary iron was negatively associated with Barrett's, i.e. decreased risk with increased intake (see Summary Table 9). This was more obvious for longer segments of Barrett. Similar was seen for Barret cases vs. GERD controls. These observations were supported by lower s-Ft and serum transferrin saturation among Barrett's Esophagus cases than other cases. Since this is a case – control study pointing in the opposite direction than the hypothesis based on animal studies, the evidence must be regarded as inconclusive (grade 4).

### Miscellaneous cancers

In a case-control study of 315 case-control pairs (study quality B) of the association between total intake of iron (diet+supplements) by mothers during the periconception period, and the occurrence of medulloblastoma/primitive neuroectodermal tumors of the brain during childhood, the adjusted OR for iron was 0.5 (95% CI: 0.3–0.9; *P*_trend_=0.008), and hence suggestive of a protective effect of high total iron intake (68). However, the authors remark that a lack of internal consistency between the findings for supplement use, intake from foods, and intake from supplements and foods combined, decrease their confidence in the results. The study must be regarded as inconclusive.

The complementary search also yielded one study on heme iron intake and the risk of bladder cancer, and one study on the association between maternal iron supply and the risk of infant leukemia (69, 70). None of the studies found any association between iron intake and cancer.

## Rheumatoid arthritis

One paper on iron intake and risk for rheumatoid arthritis was evaluated, quality grade B. The study by Benito-Garcia et al. is a prospective study on a large cohort, i.e. nurses’ health study (71). The background to this study lies in results from former studies indicating that high protein intake is involved in the etiology of rheumatoid arthritis (RA) and low protein diminished RA symptoms. The paper by Benito-Garcia et al. refer to ecological observation showing lower incidence of RA where meat intake is low, and the large European prospective investigation, EPIC, associated red meat and protein intake with risk for inflammatory polyarthritis. Therefore Benito-Garcia et al. tested the association between iron intake and RA in the large prospective Nurses health study. None of these associations with RA were found, neither for iron nor for meat or protein intake. The authors mention several possible reasons for the discrepancy between their results and those of Pattison et al. (72) for red meat and protein intake, which they did not confirm, such as different methods to collect data, FFQ vs. 7-day food diary; different origin of dietary protein in the US and European diets; different diagnosis i.e. inflammatory polyarthritis (inflammation in 2 or more joints) vs. RA verified by a screening questionnaire and medical records (72).

The results from these studies did not support the hypothesis that iron intake is associated with RA so there is no conclusive evidence for this association (grade 4).

## Gastrointestinal side effects in pregnancy

The objective of a study by Gill and co-workers (study quality C) was to determine if decreasing iron exposure in pregnancy can mitigate nausea and vomiting (73). Data were collected from a prospective cohort, Motherisk Program in Toronto. Women (*n*=97) experiencing nausea and vomiting were advised to discontinue prenatal multivitamin administration (including 27–60 mg of iron) and use instead folic acid and a usual multivitamin. The results showed that 67 or two thirds of the women reported improvement and less nausea and vomiting. The authors concluded that avoiding iron-containing supplements, in the first trimester is effective in improving nausea and vomiting in pregnancy in the majority of women suffering from morning sickness. The authors also mention that taking iron supplements in the first trimester has been considered unnecessary in most cases, which also supports avoiding extra iron intake during this time. The study cannot be used to determine total iron intake in pregnancy.

Milman et al. (study quality B) assessed gastrointestinal side effects of iron prophylaxis in pregnancy by randomizing blindly 404 healthy women in four groups receiving varying amounts of supplemental iron (20, 40, 60 or 80 mg Fe/day as ferrous fumarate), taken at bedtime (74). Side effects were recorded from an interview at 18, 32 and 39 weeks of gestation. The results showed little difference in gastrointestinal effects between the different levels of supplemental intake and compliance to supplement intake was also reported to be similar. However, constipation and black feces seemed more frequent in those taking 80 mg/day of supplemental iron (*P*<0.04 and *P*<0.001, respectively). The authors concluded that 20–80 mg/day of supplemental ferrous iron fumarate taken between meals had no clinically significant side effects and that iron prophylaxis to pregnant women should not be compromised by undue concern of side effects. Dietary intake was not assessed, so this study cannot be used to determine total iron intake in pregnancy.

The different findings and conclusions from the two studies might be due to different types of iron containing supplements, different time periods in pregnancy, i.e. first trimester vs. second and third trimesters and insufficient power. There is no conclusive evidence (grade 4) regarding the association between iron prophylaxis and gastrointestinal side effects in pregnancy.

## Maternal iron intake in pregnancy and hypospadia in male offspring

Increase in frequency of hypospadias has put forward the question of possible causes in the environment, e.g. brought to the fetus in supplements or diet. North and Golding together with the ALSPAC study team investigated the possible role of maternal diet in the origin of hypospadias in the prospective ALSPAC study (75). With special emphasis on a vegetarian diet, mainly because of a possible role of phytoestrogens in the etiology of the disease, they also found an association between hypospadias and omnivore diet supplemented with iron (OR=2.07; CI: 1–4.3). Both vegetarian diet and influenza early in pregnancy showed stronger associations with hypospadias, than found for iron supplement taken by omnivores. Of the 7,928 mothers only 163 reported to buy only organic food, but none of these had a son with hypospadias. The authors put main weight on the findings for vegetarian diet and for influenza, but dietary questions were answered in week 32. Sample size calculations and power were not reported. It is not possible to judge intake of total iron from the study, and the amount of supplemental iron is not described. Multiple testing for certain factors was made, but did not exclude by chance findings and to check for all possible confounders.

This study may indicate some association between iron supplement and hypospadias, without being able to give information about the amount and type of intake and to adjust for possible confounders. Thus, the study gives no conclusive evidence (grade 4) regarding an association between maternal iron intake in pregnancy and hypospadia in the male offspring. Further studies are needed in the area.

## Interactions between iron and other food components

Even though not covered by our initial research questions, our search generated several papers regarding interactions between iron and other food components, and we summarize the findings briefly here.

### Zinc and copper

Since copper and zinc are also divalent metals, it has been suggested that a high iron intake may competitively inhibit intestinal absorption of these two minerals, potentially leading to deficiencies. Copper is believed to use the same transporter protein as iron, divalent metal transporter 1, while zinc is believed to use specific zinc transporter proteins (76).

We included three intervention studies, all of quality A, investigating the effects of iron supplements on zinc and copper absorption or on zinc status. In the study by Domellöf et al. 25 breast-fed infants received iron supplements (1 mg/kg/day, corresponding to about 7.5 mg/day) or placebo for 2–5 months. Zinc and copper absorption were evaluated using stable isotope methods. No effect of iron supplements on zinc or copper absorption was found in this study (77). Harvey et al. randomized 13 pregnant women to receive iron supplements equaling 100 mg daily or placebo and found no significant difference in plasma zinc concentrations, exchangeable zinc pool (a measure of zinc status), urinary zinc secretion or fractional zinc absorption (78). Troost et al. investigated the effects of a single dose of 100 or 400 mg iron versus placebo in 55-year-old ileostomy subjects and found lower zinc absorption but no difference in copper absorption after iron administration (79).

The three studies are performed in very different categories of subjects and provide conflicting evidence with regard to the effect of iron supplementation on zinc and copper absorption. When excluding the study that was performed in a population less relevant to the NNR (ileostomy subjects), there is no conclusive evidence (grade 4) that iron supplements affect zinc or copper absorption.

### Tea

Two papers, both evidence quality C, have reviewed the association between tea consumption and iron status (80, 81). The two reviews have a number of overlapping references.

The first review summarizes a total of 16 studies (*N*=8,613) of which six are on infants and children (*N*=2,942), six are on premenopausal women (*N*=2,874), two are on men (*N*=1,402) and two are on the elderly (*N*=1,395) (80). Unfortunately, the review does not give any quantitative data (e.g. RR or OR) on the impact of tea consumption on iron status. Generally, the results showed that in groups with high prevalence of ID (infant/child, women) tea consumption was inversely associated with s-Ft and/or Hb. This association disappeared when adjusting for confounding dietary factors. The authors conclude that tea consumption does not influence iron status in populations having adequate iron stores. Only in individuals with marginal iron stores there may be a negative association between tea consumption and iron status.

The second review identified 36 references specific to the effects of black tea on iron absorption or iron nutrition (81). This study has a possible, non-declared conflict of interest since it is supported by PG Tips Tea, part of Unilever Bestfoods UK, and one of the authors is a consultant at Unilever Bestfoods UK. The authors found evidence that consumption of black tea inhibits iron absorption from test meals, although to a variable extent and probably less so in complete diets. When it comes to the influence of tea drinking on iron status there are indications that tea consumption in populations at risk of ID is negatively associated with iron status markers, or there are more cases of ID and IDA among tea drinkers compared to non-tea drinkers. However, the authors state that many of the studies report additional associations between iron status markers and other factors, both dietary and non-dietary, underlining the complexity of interactions determining iron absorption and iron status.

The authors conclude that there is no need to advise any restrictions on tea drinking in healthy people with no risk of ID. In groups at risk of ID, the advice should be to drink tea between meals (at least 1 h after eating)

Taken together, the evidence summarized in these two, semi-systematic reviews indicate (grade 3) that tea drinking would have a minor impact on iron nutrition in the Nordic countries, since tea drinking is not so widespread, and since most people have adequate iron stores. Only in sub-populations at increased risk of ID, should tea drinking between meals only be considered as advice.

## Mortality

During the review process of this manuscript, an additional publication from 2011 was discovered, which was not included in our original search: A cohort study of 38,772 older women in the Iowa Women's Health Study who from a mean age of 62 years were followed for 22 years and the effects of self-reported dietary supplements on mortality were investigated (82). The use of multivitamins, vitamin B6, folic acid, iron, magnesium, zinc, and copper were associated with increased risk of total mortality, however only the effects of multivitamins and copper remained significant when correcting for multiple comparisons.

## Discussion

### Anemia and iron status

#### Effects of iron intake in infants and children

Globally, it is estimated that about 25% of pre-school children have IDA (83). The prevalence of IDA in European infants is very low below the age of 6 months but increases to 2–3% at 12 months (84) and 3–7% at 1–3 years of age (85–87). In the 1990s, non-anemic ID (defined as s-Ft<12 µg/L) had a prevalence of 26–40% among 12-month-olds in the Nordic countries (88, 89) but more recent data shows a lower prevalence around 6% (90). The health consequences of non-anemic ID are less well known and the prevalence is highly dependent on the definition of ID, which is controversial during infancy (91).

Since the Hb concentration falls rapidly after birth, and iron is transferred from Hb to iron stores, the term, normal birth weight, healthy, breast-fed newborn is assumed to be virtually self-sufficient with regard to iron during the first 4–6 months of life, even though breast milk has a very low iron concentration (0.3 mg/L). Iron supplements are therefore usually not recommended for healthy, breast-fed infants. The four studies included in this review do not provide sufficient evidence that term, normal birth weight, healthy, breast-fed infants need additional dietary iron before 6 months of life in populations with a low general prevalence of IDA such as the Nordic countries.

Formula-fed infants theoretically have higher iron requirements due to the lower bioavailability of iron from formula compared to breast milk. Considering only the difference in bioavailability, an iron concentration of about 1.5 mg/L in infant formula would be sufficient but, traditionally, much higher levels of fortification have been used. In Europe, infant formulas are usually iron fortified to a level of 4–8 mg/L while American guidelines suggest 10–12 mg/L (92). The small intervention study included in this review, comparing 2 vs. 4 mg Fe/L during the first 6 months of life, does not give conclusive evidence on which iron concentration is needed in infant formula to prevent ID. Global guidelines coordinated by the European Society for Pediatric Gastroenterology, Hepatology and Nutrition (93) recommend fortification of infant formula with 0.3–1.3 mg Fe/100 kcal, corresponding to 2–8.5 mg Fe/L. We conclude that concentrations of 4–8 mg Fe/L in formulas for infants <6 months of age seems to be safe and effective based on current practice in Europe.

It is known that a low intake of iron-rich complementary foods increases the risk of ID in infants (94). Unfortunately, the studies included in this review do not give any evidence on iron requirements in infants and young children above the age of 6 months. However, one study suggested that a high intake of cow's milk (>300–500 mL/day) may be associated with ID. Therefore, it seems prudent to avoid such high intakes of cow's milk in infants and toddlers. This is supported by the reduced prevalence of ID at 12 months in Icelandic infants after new recommendations suggesting the avoidance of cow's milk before 12 months of age and the use of iron-fortified follow-on formula (90).

#### Effects of iron supplementation in fertile women

Daily iron requirement is high among women during childbearing years, because of blood loss during menstruation. This loss is variable between women, but the variation is less among adolescent girls than among adult women (95).

The daily iron intake required to meet the need of 90 and 95% of adult women is estimated to be 14.8 and 18.5 mg/day, respectively, assuming 15% absorption (see NNR4 Table 34.2). Recommended daily iron intake is 15 mg/day for women of childbearing age.

It should be emphasised that these calculations assume that blood losses in menstruation are the same as in the 1960s, although it is known that nowadays blood loss is substantially less due to the use of oral contraceptives. In a more recent study, median menstrual blood loss was 17.6 mL/day in 90 UK women of childbearing age, and median menstrual iron loss was 0.26 mg/day, with 70% of the women losing less than 0.5 mg/day (96). Approximately 30% of the women used oral contraceptives.

We found no studies examining the effect of dietary iron intake in iron-replete women of childbearing age. However, two small studies were found looking at iron status markers in women with low iron stores. In one study, an intake of between 11.5 and 13.9 mg Fe/day over 8 weeks maintained iron status independently of whether an oily fish or a red meat diet was supplied. In another study, 16 weeks intake of 500 mL/day of fruit juice supplemented with micro-encapsulated iron-pyrophosphate (total iron intake approx. 30 mg/day) increased s-Ft levels from approximately 25 to approximately 40 ng/mL, whereas fruit juice only (total iron intake approximately 15 mg/day) resulted in no increase or decrease in s-Ft levels.

Taken together, these two studies indicate that approximately 15 mg Fe/day is sufficient to maintain iron status in women with low iron stores. Supplementation with an additional 15 mg Fe/day appears sufficient to substantially improve iron status over 16 weeks. This is in accordance with NNR4, which states that 14.8 mg Fe/day covers the iron need of 90% of women in fertile age.

#### Iron supplementation in pregnancy

Maternal iron need during pregnancy increases slowly as the pregnancy progresses, because of growth and maintenance of the foetus and the uterus, increase in red blood cell count and expected iron losses when giving birth. Current Norwegian recommendations about the intake of iron supplements during pregnancy are based on measurements of s-Ft concentrations (97). Recommendations regarding dietary iron intake in pregnant women are based on the NNR 2004 recommendation for non-pregnant women. However, the physiological need of some women for iron cannot be satisfied during the last two thirds of pregnancy with foods only and supplemental iron is needed. NNR 2004, in accordance with the recommendations of SCF (98), do not give recommendations for dietary iron during pregnancy. The US recommendation for pregnancy is 27 mg/day (33).

The studies by Milman et al. (28), Makrides et al. (26), and Sandstad et al. (27) provide probable evidence (grade 2) that supplementing with 40 mg Fe/day from week 18–20 of gestation without any knowledge of s-Ft status in early pregnancy, will achieve prevention of ID in more than 90% and IDA in more than 95% of women at delivery and at 6–8 weeks postpartum. If we screen for s-Ft levels in early pregnancy (week 15–18), then 60 mg or 40 mg Fe/day will prevent ID in more than 90% and IDA in 100% at delivery in women having Ft<30 µg/L or Ft between 30 and 70 µg/L, respectively, in early pregnancy. Women having Ft>70 µg/L would normally not require iron supplementation.

There is no convincing evidence that postpartum supplementation with 80 mg Fe/day gives any lasting benefits with regard to maternal anemia.

#### Effects on offspring of iron supplementation of pregnant women

There is no convincing evidence that iron supplementation of pregnant women in a Scandinavian setting would improve iron status or cognitive development in offspring. Indeed, several high quality RCTs show a lack of such effects. A more effective method for improving the iron endowment of newborns is to practice delayed clamping of the umbilical cord at birth, which recently was shown to reduce the risk of ID at 4 months of age in Swedish infants (99).

### Physical performance

From the two studies considered, we conclude that iron supplementation does not increase physical performance in iron-normal individuals, but may have a positive effect on the performance of individuals with IDA.

### Cognitive, motoric, and behavioral function

#### Effects on the offspring of iron given to the pregnant mother

There is no evidence that iron supplementation during pregnancy improves cognitive or behavioral outcomes in the offspring in a Nordic setting.

#### Effects of iron given to infants and children

During early childhood, growth and development of the central nervous system is rapid and iron is critical for this process. Animal studies have shown that iron is essential for several aspects of brain development: Myelination, monoamine neurotransmitter function, neuronal and glial energy metabolism, and hippocampal dendritogenesis (100). Several case-control studies in children have shown a consistent association between IDA in early childhood and long-lasting poor cognitive and behavioral performance and an association between early ID and later developmental scores has been observed also in the Nordic setting (3, 101).

The study by Friel et al. (14) and the meta-analysis by Szajewska et al. (41) give limited suggestive evidence (grade 3) that preventive iron supplementation of infants may improve psychomotor development. This is supported by a previous meta-analysis by Sachdev et al. from 2005 of 17 randomised clinical trials in children (0–15 years) that included cognitive and psychomotor outcomes, which showed that iron supplementation or the intake of iron fortified foods had a significantly positive effect on mental development indices, equivalent to 1.5–2 points of 100 (102). This meta-analysis included preventive trials in risk groups, mostly in low-income countries, and therapeutic trials in children with diagnosed IDA and was thus not included in this systematic review..

However, there is insufficient evidence to conclude that preventive iron supplementation given before 6 months of life has beneficial effects on cognition or psychomotor development in healthy, breast-fed infants of normal birth weight in low-risk populations such as the Nordic countries.

Even though iron supplements are not recommended for healthy, normal birth weight infants before 6 months of age in the Nordic countries, there is one risk group that needs to be considered: Low birth weight infants. Low birth weight, defined as a birth weight <2,500 g, affects 4% of newborns in the Nordic countries and is a major risk factor for ID in children.

A meta-analysis from 1992, based on older studies, showed that prophylactic iron at a dose of 2 mg/kg/day given to LBW infants, most with birth weights 1,500–2,500 g, leads to a significantly reduced incidence of anemia at 6 months (103).

In a study by Friel et al. 58 infants with an average birth weight of 1,500 g were randomized to different infant formulas resulting in iron intakes of 3–6 vs. 2–3 mg/kg/day up to 9 months of age (104). There was no difference in anemia or neurodevelopment at 12 months. However, the high-iron group had higher glutathione peroxidase concentrations (a marker of oxidative stress), lower plasma zinc and copper levels and a higher number of respiratory tract infections, suggesting possible adverse effects with the higher iron intakes.

In a recent study, Berglund et al. randomized 285 marginally low birth weight infants (2,000–2,500 g) to receive 0, 1 or 2 mg/kg/day of iron from 6 weeks to 6 months of age and found that iron supplementation effectively prevented IDA (9.9, 2.7 and 0% respectively, *P*=0.004) and ID (36, 8 and 4%, *P*<0.001) at 6 months (105). In a follow-up of this study, it was observed that those marginally low birth weight children who were iron supplemented during early infancy had significantly lower prevalence of behavioral problems at 3 years of age (2.8 vs. 12.7%, *P*=0.027) [Berglund et al. submitted]. This supports the WHO recommendation (106) that iron supplements should be provided to all low birth weight infants, including those of marginally low birth weight, which includes moderately pre-term and term, small for gestational age infants. The Berglund study suggests that a dose of 1–2 mg/kg/day from 6 weeks to 6 months of age is a safe and effective dose in the Nordic population.

#### Possible adverse effects

One recent study (107) (retrieved in complementary search, not included in evidence tables) investigated the long term effects of an intervention in which healthy, non-anemic, Chilean infants (*N*=835) with birth weights >3 kg, were randomized to receive a high iron (12 mg/L) or low iron (2.3 mg/L) infant formula from 6–12 months of age. The initial results from this study showed no significant effect on IDA at 12 months (about 3% in both groups) (108). In a follow-up at 10 years of age (*N*=494), several dimensions of cognitive and motor development were tested and the children in the high-iron group had lower scores for all outcomes, significantly so for visual–motor integration (107). This negative effect seemed to be limited to those infants who were initially iron-replete (Hb>120 g/L). This study suggests possible adverse effects of excessive iron intake during late infancy but has several weaknesses, including a high attrition rate (41%) and needs to be confirmed by other studies before any conclusion can be drawn.

#### Effect of iron given to school children or adults

Falkingham et al. has performed a meta-analysis (study quality A) of RCTs of the effects of iron on cognitive functions when given to children aged 6–18 years, or to adult, pre-menopausal women (109). This meta-analysis by Falkingham was not included in the systematic review since it was mainly based on studies in populations not relevant for the NNR. However, the results were still interesting from a mechanistic point of view: a positive effect was found on attention/concentration (three studies) in anemic women or Indian school children at high-risk of ID. In contrast, no significant effects were observed with regard to intelligence, memory, psychomotor function or scholastic aptitude. This meta-analysis provides probable (grade 2) evidence that iron supplements improve attention and concentration in school children or adults who are anemic or who belong to a high-risk group for IDA. However, it is not clear that this is relevant to populations with a low risk of IDA, e.g. Nordic countries.

### Fetal and child growth

#### Fetal growth and pregnancy duration

We found no evidence that, in a Nordic setting, iron supplements during pregnancy improves fetal growth or reduces the proportion of children born prematurely, small for gestational age or with low birth weight.

#### Child growth

It has been suggested that iron may have adverse effects in iron-replete children. However, this review shows that there is not enough evidence to conclude that iron supplements impair growth in iron-replete children, even though more studies are needed.

## Hypertension and cardiovascular disease

### Hypertension

There is no convincing evidence for an association between high iron intake in pregnancy and lower blood pressure in the offspring (47, 48). Two studies, one prospective and one cross sectional, showed a protective effect of non-heme dietary iron consumption against higher blood pressure levels in adults (49, 50). However, the evidence is insufficient to draw a conclusion.

### Cardiovascular disease

A relation between heme iron intake and cardiovascular disease is evaluated as suggestive based on the SACN report (UK's Scientific advisory committee on nutrition treated iron and cardiovascular disease in their report on iron and health from 2010). This has also been shown in the Iowa women's health study cohort, for women having >10 g of alcohol per day and more clearly at 30 g/day (51).

## Diabetes mellitus

We found three large studies examining the association between iron intake and T2D in a total of 35,698 men (53) and 120,729 women (13, 52). The studies reported similar figures for the lowest and the highest quintile of heme iron intake, and they arrived at the exact same relative risk for T2D (RR=1.28). No effect or an inverse effect on the risk of T2D was seen for non-heme iron or for total iron intake. Thus, these studies indicate that there is an association between heme iron intake and the risk of developing T2D. Indirectly, the two case studies looking mainly at the association between markers of iron stores and risk of T2D (54, 55) supports the results above, since they found that cases with T2D also had the highest intake of heme iron.

The studies above adjusted their data for many known confounders. However, the fact that heme iron intake is closely determined by the intake of red meat, that subjects with high heme iron intake had significantly less healthy behavior (diet, physical activity, smoking, BMI) (13) and the absence of an effect of total iron intake, makes it likely that there are other dietary factors (e.g. nitrites, sodium, glycation products, inflammatory mediators) or lifestyle factors rather than iron that increase the risk of T2D.

With regard to gestational diabetes, Qiu et al. found in a study of 3,158 pregnant women that a high intake of heme iron during pregnancy increased the risk of developing GDM (56). Bowers et al. reached the same conclusion when studying the association between pre-pregnancy heme iron intake and the risk of GDM in 13,475 women (57). Thus, a high intake of heme iron both before and during pregnancy appears to significantly increase the risk of GDM.

Taking all of these studies together, we conclude that there is probable evidence (grade 2) for the association of heme iron intake with the risk of T2D and GDM even though these associations are not necessary causal. Since there is no evidence that total iron intake is associated with an increased risk of T2D, this does not have any implication for iron RDIs.

A recent meta-analysis, including data from 204,159 individuals, confirmed the association between red meat intake, particularly processed red meat, and increased risk of T2D (110). Possible health effects of meat, unrelated to iron, and recommendations regarding the intake of red or processed meat intake are beyond the scope of this paper.

The study investigating the association between iron intake and the development of T1D in infants aged 1–4 months, found an association of T1D with high total iron intake (58). The authors suggested that this was due to higher intake of iron-fortified formula, although formula intake was not recorded. An alternative explanation could be the higher intake of cow's milk proteins through formula, which seems to be associated with T1D (111). Thus, there is no conclusive evidence (grade 4) for the association of iron intake and the development of T1D.

## Cancer

According to a recent meta-analysis, individuals carrying the C282Y hemochromatosis mutation have an increased risk of hepatocellular carcinoma (112). Regarding extra-hepatic malignancies in the general population, there were some studies cited in NNR4 indicating an increased risk of colon cancer and also other cancers related to high iron stores. However, a causal relationship between iron and extra-hepatic cancer could not be established. We found a total of 12 studies examining the possible relationship between dietary iron intake and various forms of cancer.

No convincing evidence that dietary iron intake is associated with increased risk of colon cancer, lung cancer, breast cancer, esophageal cancer or other cancers, could be found.

## Rheumatoid arthritis

There is insufficient evidence to support iron intake being associated with RA, but such an association cannot be excluded. However, based on the paper by Benito-Garcia et al. (71), an exploration of prospective data having information on dietary intake, including red meat, protein and iron, and the development of RA would be valuable.

## Gastrointestinal side effects

Two studies on gastrointestinal side effects and iron intake in pregnancy found different results and no conclusions can be drawn about the association.

## Hypospadias

One study indicated some association between iron supplement and hypospadias. However, it is not possible to give information about the amount and type of intake or to adjust for possible confounders. No conclusion could be suggested. Further studies are needed in the area.

## Interaction between iron and other food components

### Copper and zinc

We included three intervention studies, all of quality A, investigating the effects of iron supplements on zinc and copper absorption or on zinc status. The three studies provide no conclusive evidence (grade 4) with regard to the effect of iron supplementation on zinc and copper absorption.

#### Tea

Tea consumption does not influence iron status in populations having adequate iron stores. We conclude that tea drinking has only a marginal impact on iron status in the Nordic countries, since tea drinking is not very widespread, and since most people have adequate iron stores. Only in sub-populations at increased risk of ID tea drinking only between meals may be considered as advice.

## The impact of ethnic multiplicity

Ethnic multiplicity has become widespread in the Nordic countries and many ethnic minority groups may have different nutrition profiles compared to the general population and some hereditary disorders, e.g. thalassemia are more common. It is difficult to cover these differences in recommendations for the population at large. Our search retrieved one study where this issue was addressed to some extent. In the SACN report from UK (46), they found that iron intakes of UK ethnic minority groups above 16 years of age were similar to the general population. The prevalence of anemia was low in men (0–4%) compared to women, ranging from 6–7% in Irish and Chinese to 29% in Indian women. Furthermore, the prevalence of ID and IDA in infants and children was unclear due to limited data on biochemical markers of iron status. Data for the Nordic countries are, to a large extent, lacking even though one study has showed a higher prevalence (20.0%) of anemia among pregnant immigrant women (of eastern Mediterranean, African, or Asian origin) compared to ethnic Danish women (4.9%) (113). We conclude that further research on this issue is needed, and we suggest that it should be addressed in the next NNR.

## Conclusions

The studies included in this systematic review provide additional evidence for the importance of dietary iron intake for different health outcomes. Most studies have focused on risk groups for ID: young children and fertile women. There is some evidence that prevention or treatment of ID and IDA improves cognitive/motor/behavioral development in young children and that treatment of IDA improves attention and concentration in school children and adult women.

With regard to newborns and infants up to 6 months of age, we conclude that:

1. There is insufficient evidence that healthy normal birth weight, exclusively breast-fed infants need additional dietary iron before 6 months of life in the Nordic countries.
2. An iron concentration of 4–8 mg/L in infant formulas seems to be safe and effective for normal birth weight infants.
3. Infants with low birth weight (<2,500 g) should receive iron supplements (1–2 mg/kg/day) during the first 6 months of age in order to prevent IDA and this may also reduce the risk of behavioral problems later on.
4. Late umbilical cord clamping should be practiced since this reduces the risk of ID in infants.

With regard to infants above 6 months of age and toddlers, we conclude that:

1. Iron-rich complementary foods (mainly meat products and iron-fortified foods) should be provided in order to achieve recommended iron intakes.
2. High intakes of non-iron-fortified cow's milk (>300–500 mL/day) should be avoided.

With regard to pregnant women, we conclude that iron supplementation from week 18–20 of gestation or earlier should be recommended. The supplementation could either be given as a general prophylaxis of 40 mg Fe/day to all, or as an individual prophylaxis of 60 or 40 mg Fe/day depending on the woman's s-Ft measured in early pregnancy (before 15 weeks of gestation). Both regimes would prevent ID and IDA at delivery and at 6–8 weeks postpartum >90 and 95% of cases, respectively (see Results). There is, however, no evidence that maternal iron supplementation has any effect on the offspring in the Nordic countries.

Overall, we did not find sufficient evidence to support a change of the iron intakes recommended in the NNR 4. However, one could consider adding recommendations for infants up to 6 months of age, low birth weight infants and pregnant women.

## Appendix

**Appendix 1:** Search terms [initial search sept 2010]

(“Iron, Dietary” [Mesh] OR
(“Iron” [Mesh] AND “Dietary Supplements” [Mesh]))

AND (“Neurobehavioral Manifestations” [Mesh] OR
“Anemia” [Mesh] OR
“Mental Processes” [Mesh] OR
“Behavior” [Mesh] OR
“Human Development” [Mesh] OR
“Growth” [Mesh] OR
“Iron Overload” [Mesh] OR
“Arthritis” [Mesh] OR
“Neoplasms” [Mesh] OR
“Cardiovascular Diseases” [Mesh] OR
Neurobehavioral Manifestations [Title/abstract] OR
Anemia [Title/abstract] OR
Mental Processes [Title/abstract] OR
Behavior [Title/abstract] OR
Human Development [Title/abstract] OR
Iron Overload [Title/abstract] OR
Arthritis [Title/abstract] OR
Cancer [Title/abstract] OR
Cardiovascular Diseases [Title/abstract] OR
“adverse effects” [Subheading])

AND (“humans” [MeSH Terms]

AND “2000/01/01” [PDAT] : “2010/08/24” [PDAT])

## Appendix

**Appendix 2:** Exclusion history

Event	No. of articles	Exclusion criteria	No. of articles excluded	No. of articles remaining
Search with final version of search criteria	1070 (abstracts)	Level 1	106	964
Second list of abstracts	964 (abstracts)	Level 2	694	270
Full-text articles received	270+1*	Level 3	221	49+1
Full-text articles for evidence table generation	49+1	Level 4	5	44+1
Full-text articles added from other sources	6	Level 1–4	2	48+1
Complementary search performed	187 (abstracts)	Level 1+2	79	108
Second round of complementary search abstracts	108	Level 3	61	47
Full-text articles ordered from complementary search	47	Level 3+4		47†
Diabetes search performed	132 (abstracts)	Level 1+2	124	8
Full-text articles ordered from diabetes search	8	Level 3+4	2	6

* This is the SACN report.

† Ar1cles were not sub2ect to 3AT evalua1on

Exclusion criteria:

1. Level 1: Study population not relevant to NNR.

2. Level 2: As Level 1+language, animal or cell studies, letters, news, congress reports.

## References

[1] DeMaeyer E, Adiels-Tegman M (1985). The prevalence of anaemia in the world. World Health Stat Q.

[2] Coad J, Conlon C (2011). Iron deficiency in women: assessment, causes and consequences. Curr Opin Clin Nutr Metab Care.

[3] Lozoff B, Beard J, Connor J, Barbara F, Georgieff M, Schallert T (2006). Long-lasting neural and behavioral effects of iron deficiency in infancy. Nutr Rev.

[4] Gunnarsson BS, Thorsdottir I, Palsson G (2005). Iron status in 6-y-old children: associations with growth and earlier iron status. Eur J Clin Nutr.

[5] Ekiz C, Agaoglu L, Karakas Z, Gurel N, Yalcin I (2005). The effect of iron deficiency anemia on the function of the immune system. Hematol J.

[6] Milman N, Pedersen P, Steig T, Melsen GV (2003). Frequencies of the hereditary hemochromatosis allele in different populations. Comparison of previous phenotypic methods and novel genotypic methods. Int J Hematol.

[7] Thorstensen K, Kvitland MA, Irgens WO, Hveem K, Asberg A (2010). Screening for C282Y homozygosity in a Norwegian population (HUNT2): the sensitivity and specificity of transferrin saturation. Scand J Clin Lab Invest.

[8] Jonsson JJ, Johannesson GM, Sigfusson N, Magnusson B, Thjodleifsson B, Magnusson S (1991). Prevalence of iron deficiency and iron overload in the adult Icelandic population. J Clin Epidemiol.

[9] Dewey KG, Domellöf M, Cohen RJ, Rivera LL, Hernell O, Lönnerdal B (2002). Iron supplementation affects growth and morbidity of breast-fed infants: results of a randomized trial in Sweden and Honduras. J Nutr.

[10] Weinberg ED (1996). The role of iron in cancer. Eur J Cancer Prev.

[11] Piperno A, Trombini P, Gelosa M, Mauri V, Pecci V, Vergani A (2002). Increased serum ferritin is common in men with essential hypertension. J Hypertens.

[12] Oppenheimer SJ (1989). Iron and infection: the clinical evidence. Acta Paediatr Scand Suppl.

[13] Lee DH, Folsom AR, Jacobs DR (2004). Dietary iron intake and type 2 diabetes incidence in postmenopausal women: the Iowa Women's Health Study. Diabetologia.

[14] Friel JK, Aziz K, Andrews WL, Harding SV, Courage ML, Adams RJ (2003). A double-masked, randomized control trial of iron supplementation in early infancy in healthy term breast-fed infants. J Pediatr.

[15] Ziegler EE, Nelson SE, Jeter JM (2009). Iron supplementation of breastfed infants from an early age. Am J Clin Nutr.

[16] Yurdakok K, Temiz F, Yalcin SS, Gumruk F (2004). Efficacy of daily and weekly iron supplementation on iron status in exclusively breast-fed infants. J Pediatr Hematol Oncol.

[17] Domellof M, Cohen RJ, Dewey KG, Hernell O, Rivera LL, Lonnerdal B (2001). Iron supplementation of breast-fed Honduran and Swedish infants from 4 to 9 months of age. J Pediatr.

[18] Hernell O, Lonnerdal B (2002). Iron status of infants fed low-iron formula: no effect of added bovine lactoferrin or nucleotides. Am J Clin Nutr.

[19] Gunnarsson BS, Thorsdottir I, Palsson G (2004). Iron status in 2-year-old Icelandic children and associations with dietary intake and growth. Eur J Clin Nutr.

[20] Heath AL, Tuttle CR, Simons MS, Cleghorn CL, Parnell WR (2002). Longitudinal study of diet and iron deficiency anaemia in infants during the first two years of life. Asia Pac J Clin Nutr.

[21] Navas-Carretero S, Perez-Granados AM, Schoppen S, Sarria B, Carbajal A, Vaquero MP (2009). Iron status biomarkers in iron deficient women consuming oily fish versus red meat diet. J Physiol Biochem.

[22] Fernandez-Gaxiola AC, De-Regil LM (2011). Intermittent iron supplementation for reducing anaemia and its associated impairments in menstruating women. Cochrane Database Syst Rev.

[23] Blanco-Rojo R, Perez-Granados AM, Toxqui L, Gonzalez-Vizcayno C, Delgado MA, Vaquero MP (2011). Efficacy of a microencapsulated iron pyrophosphate-fortified fruit juice: a randomised, double-blind, placebo-controlled study in Spanish iron-deficient women. Br J Nutr.

[24] Ruivard M, Feillet-Coudray C, Rambeau M, Gerbaud L, Mazur A, Rayssiguier Y (2006). Effect of daily versus twice weekly long-term iron supplementation on iron absorption and status in iron-deficient women: a stable isotope study. Clin Biochem.

[25] Milman N, Bergholt T, Eriksen L, Byg KE, Graudal N, Pedersen P (2005). Iron prophylaxis during pregnancy – how much iron is needed? A randomized dose- response study of 20–80 mg ferrous iron daily in pregnant women. Acta Obstet Gynecol Scand.

[26] Makrides M, Crowther CA, Gibson RA, Gibson RS, Skeaff CM (2003). Efficacy and tolerability of low-dose iron supplements during pregnancy: a randomized controlled trial. Am J Clin Nutr.

[27] Sandstad B, Borch-Iohnsen B, Andersen GM, Dahl-Jørgensen B, Frøysa I, Leslie C (2003). Selective iron supplementation based on serum ferritin values early in pregnancy: are the Norwegian recommendations satisfactory?. Acta Obstet Gynecol Scand.

[28] Milman N, Byg KE, Bergholt T, Eriksen L, Hvas AM (2006). Body iron and individual iron prophylaxis in pregnancy – should the iron dose be adjusted according to serum ferritin?. Ann Hematol.

[29] Cogswell ME, Parvanta I, Ickes L, Yip R, Brittenham GM (2003). Iron supplementation during pregnancy, anemia, and birth weight: a randomized controlled trial. Am J Clin Nutr.

[30] Meier PR, Nickerson HJ, Olson KA, Berg RL, Meyer JA (2003). Prevention of iron deficiency anemia in adolescent and adult pregnancies. Clin Med Res.

[31] Mara M, Zivny J, Eretova V, Kvasnicka J, Kuzel D, Umlaufova A (2001). Changes in markers of anemia and iron metabolism and how they are influenced by antianemics in postpartum period. Acta Obstet Gynecol Scand.

[32] Andersen NL, Fagt S, Groth MV, Hartkopp HB, Möller A, Ovesen L (1995). Dietary habits in Denmark 1995. Main results.

[33] Institute of Medicine (2001). Dietary reference intakes for vitamin A, vitamin K, arsenic, boron, chromium, copper, iodine, iron, molybdenum, nickel, silicon, vanadium and zinc.

[34] Pena-Rosas JP, Viteri FE (2009). Effects and safety of preventive oral iron or iron+folic acid supplementation for women during pregnancy. Cochrane Database Syst Rev.

[35] Zhou SJ, Gibson RA, Makrides M (2007). Routine iron supplementation in pregnancy has no effect on iron status of children at six months and four years of age. J Pediatr.

[36] Preziosi P, Prual A, Galan P, Daouda H, Boureima H, Hercberg S (1997). Effect of iron supplementation on the iron status of pregnant women: consequences for newborns. Am J Clin Nutr.

[37] McClung JP, Karl JP, Cable SJ, Williams KW, Nindl BC, Young AJ (2009). Randomized, double-blind, placebo-controlled trial of iron supplementation in female soldiers during military training: effects on iron status, physical performance, and mood. Am J Clin Nutr.

[38] Tsalis G, Nikolaidis MG, Mougios V (2004). Effects of iron intake through food or supplement on iron status and performance of healthy adolescent swimmers during a training season. Int J Sports Med.

[39] Zhou SJ, Gibson RA, Crowther CA, Baghurst P, Makrides M (2006). Effect of iron supplementation during pregnancy on the intelligence quotient and behavior of children at 4 y of age: long-term follow-up of a randomized controlled trial. Am J Clin Nutr.

[40] Parsons AG, Zhou SJ, Spurrier NJ, Makrides M (2008). Effect of iron supplementation during pregnancy on the behaviour of children at early school age: long-term follow-up of a randomised controlled trial. Br J Nutr.

[41] Szajewska H, Ruszczynski M, Chmielewska A (2010). Effects of iron supplementation in nonanemic pregnant women, infants, and young children on the mental performance and psychomotor development of children: a systematic review of randomized controlled trials. Am J Clin Nutr.

[42] Lind T, Lönnerdal B, Stenlund H, Gamayanti IL, Ismail D, Seswandhana R (2004). A community-based randomized controlled trial of iron and zinc supplementation in Indonesian infants: effects on growth and development. Am J Clin Nutr.

[43] Moffatt ME, Longstaffe S, Besant J, Dureski C (1994). Prevention of iron deficiency and psychomotor decline in high-risk infants through use of iron-fortified infant formula: a randomized clinical trial. J Pediatr.

[44] Baker PN, Wheeler SJ, Sanders TA, Thomas JE, Hutchinson CJ, Clarke K (2009). A prospective study of micronutrient status in adolescent pregnancy. Am J Clin Nutr.

[45] Ramakrishnan U, Nguyen P, Martorell R (2009). Effects of micronutrients on growth of children under 5 y of age: meta-analyses of single and multiple nutrient interventions. Am J Clin Nutr.

[46] SACN (2010). Iron and health (Scientific Advisory Committee on Nutrition of the UK Department of Health).

[47] Belfort MB, Rifas-Shiman SL, Rich-Edwards JW, Kleinman KP, Oken E, Gillman MW (2008). Maternal iron intake and iron status during pregnancy and child blood pressure at age 3 years. Int J Epidemiol.

[48] Brion MJ, Leary SD, Smith GD, McArdle HJ, Ness AR (2008). Maternal anemia, iron intake in pregnancy, and offspring blood pressure in the Avon Longitudinal Study of parents and children. Am J Clin Nutr.

[49] Galan P, Vergnaud AC, Tzoulaki I, Buyck JF, Blacher J, Czernichow S (2010). Low total and nonheme iron intakes are associated with a greater risk of hypertension. J Nutr.

[50] Tzoulaki I, Brown IJ, Chan Q, Van Horn L, Ueshima H, Zhao L (2008). Relation of iron and red meat intake to blood pressure: cross sectional epidemiological study. BMJ.

[51] Lee DH, Folsom AR, Jacobs DR (2005). Iron, zinc, and alcohol consumption and mortality from cardiovascular diseases: the Iowa Women's Health Study. Am J Clin Nutr.

[52] Rajpathak S, Ma J, Manson J, Willett WC, Hu FB (2006). Iron intake and the risk of type 2 diabetes in women: a prospective cohort study. Diabetes Care.

[53] Jiang R, Ma J, Ascherio A, Stampfer MJ, Willett WC, Hu FB (2004). Dietary iron intake and blood donations in relation to risk of type 2 diabetes in men: a prospective cohort study. Am J Clin Nutr.

[54] Jiang R, Manson JE, Meigs JB, Ma J, Rifai N, Hu FB (2004). Body iron stores in relation to risk of type 2 diabetes in apparently healthy women. JAMA.

[55] Forouhi NG, Harding AH, Allison M, Sandhu MS, Welch A, Luben R (2007). Elevated serum ferritin levels predict new-onset type 2 diabetes: results from the EPIC-Norfolk prospective study. Diabetologia.

[56] Qiu C, Zhang C, Gelaye B, Enquobahrie DA, Frederick IO, Williams MA (2011). Gestational diabetes mellitus in relation to maternal dietary heme iron and nonheme iron intake. Diabetes Care.

[57] Bowers K, Yeung E, Williams MA, Qi L, Tobias DK, Hu FB (2011). A prospective study of prepregnancy dietary iron intake and risk for gestational diabetes mellitus. Diabetes Care.

[58] Ashraf AP, Eason NB, Kabagambe EK, Haritha J, Meleth S, McCormick KL (2010). Dietary iron intake in the first 4 months of infancy and the development of type 1 diabetes: a pilot study. Diabetol Metab Syndr.

[59] Lee DH, Anderson KE, Harnack LJ, Folsom AR, Jacobs DR (2004). Heme iron, zinc, alcohol consumption, and colon cancer: Iowa Women's Health Study. J Natl Cancer Inst.

[60] Chan AT, Ma J, Tranah GJ, Giovannucci EL, Rifai N, Hunter DJ (2005). Hemochromatosis gene mutations, body iron stores, dietary iron, and risk of colorectal adenoma in women. J Natl Cancer Inst.

[61] Bastide NM, Pierre FH, Corpet DE (2011). Heme iron from meat and risk of colorectal cancer: a meta-analysis and a review of the mechanisms involved. Cancer Prevent Res (Phila).

[62] Lee DH, Jacobs DR (2005). Interaction among heme iron, zinc, and supplemental vitamin C intake on the risk of lung cancer: Iowa Women's Health Study. Nutr Cancer.

[63] Zhou W, Park S, Liu G, Miller DP, Wang LI, Pothier L (2005). Dietary iron, zinc, and calcium and the risk of lung cancer. Epidemiology.

[64] Tasevska N, Cross AJ, Dodd KW, Ziegler RG, Caporaso NE, Sinha R (2011). No effect of meat, meat cooking preferences, meat mutagens or heme iron on lung cancer risk in the prostate, lung, colorectal and ovarian cancer screening trial. Int J Cancer.

[65] Ferrucci LM, Cross AJ, Graubard BI, Brinton LA, McCarty CA, Ziegler RG (2009). Intake of meat, meat mutagens, and iron and the risk of breast cancer in the prostate, lung, colorectal, and ovarian cancer screening trial. Br J Cancer.

[66] Kabat GC, Cross AJ, Park Y, Schatzkin A, Hollenbeck AR, Rohan TE (2010). Intakes of dietary iron and heme-iron and risk of postmenopausal breast cancer in the National Institutes of Health-AARP Diet and Health Study. Am J Clin Nutr.

[67] Corley DA, Kubo A, Levin TR, Habel L, Zhao W, Leighton P (2008). Iron intake and body iron stores as risk factors for Barrett's esophagus: a community-based study. Am J Gastroenterol.

[68] Bunin GR, Kushi LH, Gallagher PR, Rorke-Adams LB, McBride ML, Cnaan A (2005). Maternal diet during pregnancy and its association with medulloblastoma in children: a children's oncology group study (United States). Cancer Causes Control.

[69] Jakszyn P, Gonzalez CA, Lujan-Barroso L, Ros MM, Buenode- Mesquita HB, Roswall N (2011). Red meat, dietary nitrosamines, and heme iron and risk of bladder cancer in the European Prospective Investigation into Cancer and Nutrition (EPIC). Cancer Epidemiol Biomarkers Prev.

[70] Linabery AM, Puumala SE, Hilden JM, Davies SM, Heerema NA, Roesler MA (2010). Maternal vitamin and iron supplementation and risk of infant leukaemia: a report from the Children's Oncology Group. Br J Cancer.

[71] Benito-Garcia E, Feskanich D, Hu FB, Mandl LA, Karlson EW (2007). Protein, iron, and meat consumption and risk for rheumatoid arthritis: a prospective cohort study. Arthritis Res Ther.

[72] Pattison DJ, Symmons DP, Lunt M, Welch A, Luben R, Bingham SA (2004). Dietary risk factors for the development of inflammatory polyarthritis: evidence for a role of high level of red meat consumption. Arthritis Rheum.

[73] Gill SK, Maltepe C, Koren G (2009). The effectiveness of discontinuing iron-containing prenatal multivitamins on reducing the severity of nausea and vomiting of pregnancy. J Obstet Gynaecol.

[74] Milman N, Byg KE, Bergholt T, Eriksen L (2006). Side effects of oral iron prophylaxis in pregnancy – myth or reality?. Acta Haematol.

[75] North K, Golding J (2000). A maternal vegetarian diet in pregnancy is associated with hypospadias. The ALSPAC Study Team. Avon Longitudinal Study of Pregnancy and Childhood. BJU Int.

[76] Lönnerdal B (2005). Trace element nutrition of infants – molecular approaches. J Trace Elem Med Biol.

[77] Domellof M, Hernell O, Abrams SA, Chen Z, Lonnerdal B (2009). Iron supplementation does not affect copper and zinc absorption in breastfed infants. Am J Clin Nutr.

[78] Harvey LJ, Dainty JR, Hollands WJ, Bull VJ, Hoogewerff JA, Foxall RJ (2007). Effect of high-dose iron supplements on fractional zinc absorption and status in pregnant women. Am J Clin Nutr.

[79] Troost FJ, Brummer RJ, Dainty JR, Hoogewerff JA, Bull VJ, Saris WH (2003). Iron supplements inhibit zinc but not copper absorption in vivo in ileostomy subjects. Am J Clin Nutr.

[80] Temme EH, Van Hoydonck PG (2002). Tea consumption and iron status. Eur J Clin Nutr.

[81] Nelson M, Poulter J (2004). Impact of tea drinking on iron status in the UK: a review. J Hum Nutr Diet.

[82] Mursu J, Robien K, Harnack LJ, Park K, Jacobs DR (2011). Dietary supplements and mortality rate in older women: the Iowa Women's Health Study. Arch Intern Med.

[83] McLean E, Cogswell M, Egli I, Wojdyla D, de Benoist B (2009). Worldwide prevalence of anaemia, WHO Vitamin and Mineral Nutrition Information System, 1993–2005. Public Health Nutr.

[84] Male C, Persson LA, Freeman V, Guerra A, van't Hof MA, Haschke F (2001). Prevalence of iron deficiency in 12-mo-old infants from 11 European areas and influence of dietary factors on iron status (Euro-Growth study). Acta Paediatr.

[85] Thane CW, Walmsley CM, Bates CJ, Prentice A, Cole TJ (2000). Risk factors for poor iron status in British toddlers: further analysis of data from the National Diet and Nutrition Survey of children aged 1.5–4.5 years. Public Health Nutr.

[86] Hay G, Sandstad B, Whitelaw A, Borch-Iohnsen B (2004). Iron status in a group of Norwegian children aged 6–24 months. Acta Paediatr.

[87] Bramhagen A-C, Axelsson I (1999). Iron status of children in southern Sweden: effects of cow's milk and follow-on formula. Acta Paediatr.

[88] Persson LÅ, Lundström M, Lönnerdal B, Hernell O (1998). Are weaning foods causing impaired iron and zinc status in 1-year-old Swedish infants? A cohort study. Acta Paediatr.

[89] Thorsdottir I, Gunnarsson BS, Atladottir H, Michaelsen KF, Palsson G (2003). Iron status at 12 months of age – effects of body size, growth and diet in a population with high birth weight. Eur J Clin Nutr.

[90] Thorisdottir AV, Thorsdottir I, Palsson GI (2011). Nutrition and iron status of 1-year olds following a revision in infant dietary recommendations. Anemia.

[91] Domellöf M, Dewey KG, Lönnerdal B, Cohen RJ, Hernell O (2002). The diagnostic criteria for iron deficiency in infants should be re-evaluated. J Nutr.

[92] Domellof M (2011). Iron requirements in infancy. Ann Nutr Metab.

[93] Koletzko B, Baker S, Cleghorn G, Neto UF, Gopalan S, Hernell O (2005). Global standard for the composition of infant formula: recommendations of an ESPGHAN coordinated international expert group. J Pediatr Gastroenterol Nutr.

[94] Agostoni C, Decsi T, Fewtrell M, Goulet O, Kolacek S, Koletzko B (2008). Complementary feeding: a commentary by the ESPGHAN Committee on Nutrition. J Pediatr Gastroenterol Nutr.

[95] Hallberg L, Rossander-Hulten L (1991). Iron requirements in menstruating women. Am J Clin Nutr.

[96] Harvey LJ, Armah CN, Dainty JR, Foxall RJ, John Lewis D, Langford NJ (2005). Impact of menstrual blood loss and diet on iron deficiency among women in the UK. Br J Nutr.

[97] Borch-Iohnsen B, Halvorsen R, Andrew M, Forde R, Matheson I, Rytter E (1993). Do we need new guidelines on iron supplementation during pregnancy?. Tidsskr Nor Laegeforen.

[98] Commission of the European Communities (1993). Reports of the Scientific Committee for Food: nutrient and energy intakes for the European Community.

[99] Andersson O, Hellstrom-Westas L, Andersson D, Domellof M (2011). Effect of delayed versus early umbilical cord clamping on neonatal outcomes and iron status at 4 months: a randomised controlled trial. BMJ.

[100] Carlson ES, Tkac I, Magid R, O'Connor MB, Andrews NC, Schallert T (2009). Iron is essential for neuron development and memory function in mouse hippocampus. J Nutr.

[101] Gunnarsson BS, Thorsdottir I, Palsson G, Gretarsson SJ (2007). Iron status at 1 and 6 years versus developmental scores at 6 years in a well-nourished affluent population. Acta Paediatr.

[102] Sachdev H, Gera T, Nestel P (2005). Effect of iron supplementation on mental and motor development in children: systematic review of randomised controlled trials. Public Health Nutr.

[103] Doyle JJ, Zipursky A, Sinclair JC, Bracken MB (1992). Neonatal blood disorders. Effective care of the newborn infant.

[104] Friel JK, Andrews WL, Aziz K, Kwa PG, Lepage G, L'Abbe MR (2001). A randomized trial of two levels of iron supplementation and developmental outcome in low birth weight infants. J Pediatr.

[105] Berglund S, Westrup B, Domellof M (2010). Iron supplements reduce the risk of iron deficiency anemia in marginally low birth weight infants. Pediatrics.

[106] WHO (2001). Iron deficiency anemia: assessment, prevention and control.

[107] Lozoff B, Castillo M, Clark KM, Smith JB (2012). Iron-fortified vs low-iron infant formula: developmental outcome at 10 years. Arch Pediatr Adolesc Med.

[108] Walter T, Pino P, Pizarro F, Lozoff B (1998). Prevention of iron-deficiency anemia: comparison of high- and low-iron formulas in term healthy infants after six months of life. J Pediatr.

[109] Falkingham M, Abdelhamid A, Curtis P, Fairweather-Tait S, Dye L, Hooper L (2010). The effects of oral iron supplementation on cognition in older children and adults: a systematic review and meta-analysis. Nutr J.

[110] Pan A, Sun Q, Bernstein AM, Schulze MB, Manson JE, Willett WC (2011). Red meat consumption and risk of type 2 diabetes: 3 cohorts of US adults and an updated meta-analysis. Am J Clin Nutr.

[111] Knip M, Virtanen SM, Akerblom HK (2010). Infant feeding and the risk of type 1 diabetes. Am J Clin Nutr.

[112] Jin F, Qu LS, Shen XZ (2010). Association between C282Y and H63D mutations of the HFE gene with hepatocellular carcinoma in European populations: a meta-analysis. J Exp Clin Cancer Res.

[113] Nybo M, Friis-Hansen L, Felding P, Milman N (2007). Higher prevalence of anemia among pregnant immigrant women compared to pregnant ethnic Danish women. Ann Hematol.

